# Different Strokes for Different Croaks: Using an African Reed Frog Species Complex as a Model to Understand Idiosyncratic Population Requirements for Conservation Management

**DOI:** 10.1111/eva.70164

**Published:** 2025-10-02

**Authors:** Christopher D. Barratt, Beryl A. Bwong, Lucinda P. Lawson, John V. Lyakurwa, Sebastian Steinfartz, Hendrik Müller, Robert Jehle, Simon P. Loader

**Affiliations:** ^1^ German Centre for Integrative Biodiversity Research (iDiv) Leipzig Germany; ^2^ University of Leipzig Leipzig Germany; ^3^ Naturalis Biodiversity Center Leiden the Netherlands; ^4^ Animal Breeding and Genomics Wageningen University and Research Wageningen the Netherlands; ^5^ National Museums of Kenya Nairobi Kenya; ^6^ University of Cincinnati Cincinnati Ohio USA; ^7^ Cincinnati Children's Hospital and Medical Center Cincinnati Ohio USA; ^8^ University of Dar es Salaam Dar es Salaam Tanzania; ^9^ Central Natural Science Collections Martin Luther University Halle‐Wittenberg Halle Germany; ^10^ Natural History Museum London UK; ^11^ University of Salford Salford UK

**Keywords:** adaptation, biodiversity hotspot, climate change, coastal forests of Eastern Africa, Eastern Afromontane, global change, predictive models

## Abstract

Biodiversity is under increasing pressure from environmental change, although the scope and severity of these impacts remain incompletely understood. For many species, a lack of information about population‐specific responses to future environmental change hinders the development of effective conservation strategies. Here, we use an East African reed frog species complex as a model to explore spatial variation in vulnerability to future environmental changes. Our sampling across two threatened biodiversity hotspots spans the entire geographic range of *H. mitchelli and H. rubrovermiculatus* in Kenya, Tanzania, and Malawi. Using genome‐wide (ddRAD‐seq) data, we evaluate levels of neutral genetic diversity and local adaptations across sampling localities. We then integrate spatial approaches (genomic offset, modeled dispersal barriers, and Species Distribution Models) to predict how populations may respond differently to future environmental changes, such as climate warming and predicted land use changes. Based on our analyses, we characterize population structure and identify region‐specific management needs that reflect genetic variation among populations and the uneven impacts of predicted change across the landscape. Peripheral populations are most vulnerable to future environmental changes due to (i) low levels of neutral genetic diversity (Malawi and Pare mountains in Tanzania), (ii) putative signals of local adaptation to wetter conditions with predicted disruptions to genotype–environment associations (i.e., high genomic offset, Kenya and Northern Tanzania), and (iii) the projected contraction of suitable habitat, which is a pervasive threat to the species complex in general. Populations in Northern, Central, and Southern Tanzania show the lowest vulnerability to environmental change and may serve as important reservoirs of genetic diversity for potential future genetic rescue initiatives. Our study highlights how populations across different parts of species ranges may be unevenly affected by future global changes and provides a framework to predict which conservation actions may help mitigate these effects.

## 1 Introduction

Halting catastrophic biodiversity loss in the face of global change is a central tenet of conservation. In addition to the natural fragmentation of species ranges due to long‐term climatic processes (e.g., glacial and interglacial cycles), the distribution of biodiversity across the Earth has become further fragmented due to a combination of human‐induced habitat destruction and more recent climate change (Haddad et al. 2015; Frankham et al. 2019). However, the extent, intensity, and conservation implications of this remain incompletely understood across most species' ranges (Wilson et al. 2016; Fahrig 2017; Fletcher et al. 2018; IPCC 2022). Providing information about spatial variation in the effects of global change improves our ability to predict which management strategies may be most suitable across different parts of species' ranges (Aguirre‐Liguori et al. 2021; Urban et al. 2024).

### 1.1 Global Change Impacts Vary Across Geographic Space

Species with spatially fragmented geographic distributions (either metapopulations that exhibit gene flow between demes or non‐interacting spatially isolated populations) offer a substantial challenge for conservation efforts, as these often represent a mixture of different conservation requirements. This is particularly true in under‐sampled tropical regions, where comprehensive spatial sampling often reveals structured populations due to the legacy of past climatic processes (e.g., Vieites et al. 2009; Funk, Caminer, and Santiago 2012; Barratt, Bwong, et al. 2017). Such fragmented distributions often correlate with vulnerability to local extinctions in vertebrates (Crooks et al. 2017) and amphibians in particular (Cushman 2006), outlining the need for conservation assessments that provide information below the species level. Amplifying the effects of past climate changes on already fragmented species ranges, land use change (including habitat fragmentation and destruction) is a major driver of recent biodiversity declines worldwide (IPBES 2019). Diminished habitat quantity and quality hinders gene flow and can reduce effective population size, leading to the erosion of standing genetic diversity through genetic drift and an accumulation of deleterious mutations through inbreeding, potentially resulting in fitness reductions (Frankham 1995; van Oosterhout 2020; Bertorelle et al. 2022; Dussex et al. 2023). In addition, rapid climate change and extreme weather events can drastically alter local environmental conditions, potentially disrupting local genotype–environment associations that have evolved in populations over longer periods of time (Fitzpatrick and Keller 2015). Combined with reduced standing genetic diversity, this may lead to maladaptation of populations to their local environment (Carlson et al. 2014) and, in the worst case, local extirpation.

In conservation planning for spatially structured populations, it is often assumed that small, isolated populations are the most vulnerable to local extinction due to the accentuated effects of climate change and habitat fragmentation (Lynch et al. 1995; Frankham 2015; Willi et al. 2006, 2013). However, larger and highly connected populations may also be at risk of declines, but are underestimated when ignoring metapopulation effects (Higgins and Lynch 2001). A growing body of work suggests that metapopulation structure with significant gene flow and high genome‐wide genetic diversity is likely key to maintaining the ecological resilience of smaller populations (Hanski and Simberloff 1997; Mills 2012; Lawson et al. 2019; Kardos et al. 2021). Thus, larger populations should also be considered in conservation decisions, as they safeguard smaller populations against the negative impacts of habitat fragmentation and climate change (Hoffmann et al. 2021).

Underpinning intraspecific conservation efforts at the intraspecific level is the consideration of population structure and the delineation of boundaries or zones of gene flow among populations (Ryder 1986; Moritz 1994; Avise 2004; Funk, McKay, et al. 2012; Hoelzel 2023; Turbek et al. 2023). In addition to neutral population structure, adaptive differences across populations (i.e., differential local adaptations to the environment) must also be accounted for when making management decisions (Funk, McKay, et al. 2012; Razgour et al. 2019; Aguirre‐Liguori et al. 2021; Forester et al. 2022). In response to years of neglecting genetic diversity in conservation policy (Hoban et al. 2021), global initiatives, such as the Convention on Biological Diversity (CBD), now specifically target the maintenance of genetic diversity of wild and domesticated species to minimize the risk of extirpation and extinction (CBD 2022a). The global biodiversity framework, for example, now includes a genetic indicator focusing on the proportion of populations within species with an effective population size > 500 (CBD 2022b; Mastretta‐Yanes et al. 2024).

### 1.2 African Amphibians as a Model System to Understand Idiosyncratic Population Management Requirements

Africa, with its rich biodiversity, growing human population (approaching 2.5 billion by 2050, World Bank 2024), and rapid climate change predictions (between 2°C and 3°C, IPCC 2022) is a continent facing exceptional future challenges (Chapman et al. 2022; IPCC 2023). Rich in biodiversity and supporting the largest number of threatened species of any land region (*n* = 11,043, 24% globally, IUCN 2023), sub‐Saharan Africa is home to almost a fifth of the world's biodiversity hotspots (Myers et al. 2000). A number of ecoregions, particularly in Eastern and Western Africa, are regarded as ‘Vulnerable’, ‘Endangered’, or ‘Threatened’ (Brooks et al. 2002; Burgess et al. 2004), and are priority regions for predicting the effects of future global change on biodiversity and for mitigating these with relevant conservation actions.

Amphibians, due to their high species numbers and intraspecific diversity, and sensitivity to changes in their environment, are a highly suitable model to predict the effects of global change (Cushman 2006; Wake and Vredenburg 2008; Luedtke et al. 2023; Pottier et al. 2025). In sub‐Saharan Africa, there are 1169 recognized species, 33% of which (*n* = 372) are listed as threatened (IUCN 2023). In the last decade, descriptions from sub‐Saharan African biodiversity hotspots and their surrounding landscapes of new species (e.g., Loader et al. 2015; Barratt, Lawson, et al. 2017; Conradie et al. 2018; Lawson et al. 2023; Malonza and Wasonga 2023; Griesbaum et al. 2023; Malonza 2023; du Preez et al. 2024), new genera (Nečas et al. 2021; Liedtke et al. 2023), and even entire new families (Barej et al. 2014) have enhanced our understanding of taxonomy and systematics. However, due to incomplete sampling across most species ranges, our understanding of population‐level intraspecific diversity and associated conservation needs remains limited to only a few taxa. Novel genomic technologies over the past decade have led to improved insights about the systematics and population genetics of several widespread amphibian species in this region, generally revealing high population structuring and varying demographic histories across populations due to the combined effects of geology, historical forest dynamics, climatic and riverine barriers (Bell et al. 2015; Portik et al. 2017; Barratt et al. 2018; Charles et al. 2018; Reyes‐Velasco, Manthey, Bourgeois, et al. 2018; Reyes‐Velasco, Manthey, Freilich, and Boissinot 2018; Leaché et al. 2019; Bwong et al. 2020; Jaynes et al. 2022; Miller et al. 2024; Lawson et al. 2025).

Building on these data, predicting how populations may respond to future changes is a crucial next step for evidence‐based conservation (Capblancq et al. 2020; Capblancq and Forester 2021; Aguirre‐Liguori et al. 2021; Bay et al. 2018; Forester et al. 2023; Razgour et al. 2019; Ruegg et al. 2018; Barratt, Onstein, et al. 2024; Barratt, Preißler, et al. 2024; Carneiro et al. 2025). To this end, creating an integrative understanding of how ecological and evolutionary processes may be affected by future environmental changes is imperative for effective predictive modeling. For example, across different parts of a species range, are populations able to stay in place or migrate as a response to changes in their environment? And if they are unable to migrate to track suitable environmental conditions, do populations possess sufficient genetic diversity and evolutionary potential to adapt to these changes in the short‐ and long‐term? Addressing these knowledge shortfalls with empirical data and real‐world examples will improve our ability to make informed decisions about how best to implement conservation efforts.

In this work, we combine spatial and genomic evidence to help formulate and direct conservation strategies in an East African reed frog species complex ( *Hyperolius mitchelli* and *H. rubrovermiculatus* ), hereafter referred to as the *H. mitchelli* complex (Conradie et al. 2018; Bwong et al. 2020). We use this species complex, distributed across two biodiversity hotspots (the Eastern Afromontane Region and the Coastal Forests of Eastern Africa) in Kenya, Tanzania, and Malawi, as a model system to predict how forthcoming global change effects may vary across geographic space. Given our evidence‐based approach, we evaluate which management strategies are most appropriate to mitigate these predictions. *Hyperolius mitchelli* , Loveridge 1953, and *H. rubrovermiculatus* , Schiøtz 1975 (the latter restricted to the Shimba Hills and surrounding areas in Kenya), share a recent common ancestor (estimated 2.72 mya divergence, Portik et al. 2019) but are clearly distinct from one another in terms of their coloration and geographic distributions. *Hyperolius mitchelli* , throughout its wide range in Tanzania and Malawi, demonstrates a wide variety of color patterning, mainly with a brownish dorsum and yellow to orange ventral surface, whereas *H. rubrovermiculatus* tends to have a darker colored dorsum with red vermiculations. Both *H. mitchelli* and *H. rubrovermiculatus* possess a characteristic light spot on the heel. Previous studies identified phylogeographic structure within this complex (Barratt, Bwong, et al. 2017; Bwong et al. 2020), but incomplete spatial sampling and mitochondrial‐only (matrilineally inherited) data sets left uncertainty in the conservation implications. *Hyperolius mitchelli* is listed as Least Concern on the IUCN red list, whereas *H. rubrovermiculatus* is listed as Endangered (IUCN 2023). Using newly collected field samples, environmental, and high‐resolution genome‐wide ddRAD‐seq data, we ask: (i) Does population genetic structure exist? If so, how is it distributed geographically and are there natural barriers to gene flow? (ii) What levels of genetic diversity, including effective population size, exist within each population? (iii) Which genomic regions are involved in local adaptation to climate, and how are these local adaptations distributed among populations across geographic space? and (iv) Which parts of the geographic range are most at risk of local extirpation based on the predicted effects of future global change, in terms of their genetic composition (genomic offset), and predicted habitat suitability and range shifts?

## 2 Methods and Materials

### 2.1 Sampling, Genomic Library Preparation, and Data Processing

**FIGURE 1:**
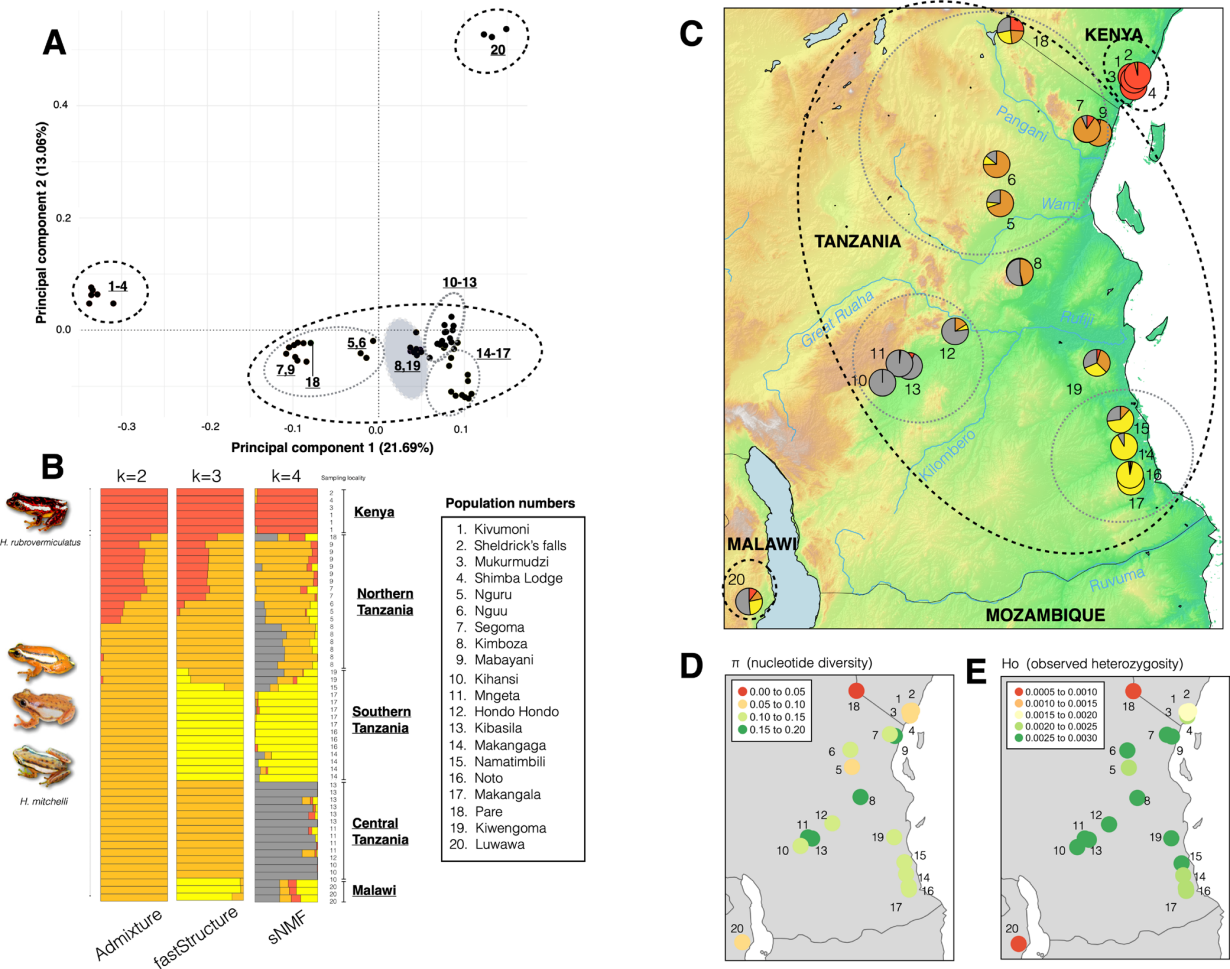
Population structure and genetic diversity across 20 sampling localities of *H. mitchelli* and *H. rubrovermiculatus* . (A) Population structure based on PCA in PLINK. (B) Admixture (*k* = 2), fastStructure (*k* = 3) and corresponding sNMF (*k* = 4) analyses, geography of sampling localities is noted to the right of the barplots, and a key to sampling localities on maps (1–20) is shown. (C) Spatial visualization of sNMF population structure at *k* = 4, colors of clusters in pie charts at each sampling locality matching sNMF outputs. The three major population clusters identified across analyses are demarcated (thicker dotted polygons), with the population substructure in Tanzania demarcated with thinner dotted polygons. In the PCA (A), localities 8 and 19 are shaded in a grey ellipse due to their unclear position between Northern, Central and Southern Tanzania. Major rivers and mountains are shown on the map. (D) Nucleotide diversity (*π*) and (E) Observed heterozygosity (*H*_o_) calculated across sampling localities based on 3849 unlinked genome‐wide SNPs (red = low, green = high).

Between 2009 and 2023, *H. mitchelli* complex reed frog leg muscle tissue (adults) and tail fin clips (tadpoles) were collected at 24 sampling localities across Kenya, Tanzania, and Malawi (Figure 1). Tissue samples were stored at room temperature after collection in 96% ethanol at the Natural History Museum (London, UK), the Museo delle Scienze (MUSE; Trento, Italy), the Field Museum of Natural History (Chicago, USA), and the Museum of Comparative Zoology (Harvard, USA). Genomic DNA was extracted for a total of 115 individuals using the DNeasy Blood & Tissue Kit (Qiagen). DNA isolates were quantified using a Qubit fluorometer (Invitrogen), equalized to a working concentration of 10 ng/μL when possible, and stored at −20°C. Retaining all samples that met this DNA working concentration to prepare genomic libraries resulted in 55 individuals across 20 unique sampling localities across Kenya, Tanzania and Malawi (ranging between 1 and 6 individuals per locality, see Table S1). Double digest RAD‐seq (ddRAD‐seq, Peterson et al. 2012) libraries were prepared by LGC Genomics (Berlin, Germany) using *PstI* and *ApekI* restriction enzymes, barcoding individual samples, and paired‐end sequencing (2 × 150 bp) on a single lane of a NovaSeq6000.

We used Stacks v.2.62 (Rochette et al. 2019) to process ddRAD‐seq data and produce SNP data sets, using the *process_radtags* module to demultiplex individuals based on their individual barcodes. Following best practices (Paris et al. 2017) for Stacks, we first explored our data set using a subset of individuals (*n* = 8) from across our geographic sampling to optimize parameter settings before further analyses (see Figure S1 for a summary of data exploration). As sequencing effort across samples was relatively even (see Results), we ran *denovo_map.pl* with all samples using our optimized parameters of *m* = 5 and *M* = 2, retaining only a single SNP per RAD locus, and only present in 80% of all individuals processed. For all downstream bioinformatic analyses, specific output file formats (*.map, *.ped, *.raw, *.vcf) as required for each analysis were generated by iteratively rerunning the *populations* module of Stacks. To verify that there was no bias in our combined SNP dataset for both species, we performed an additional SNP discovery step on each species separately using the same pipeline but without applying a minor allele frequency (MAF) filter. This allowed us to compare the extent of species‐specific vs. shared SNPs and to evaluate how much of the diversity in each species is represented in the final filtered SNP set used in our analyses (3849 SNPs, filtered at MAF > 0.05). The procedure and results for this can be found in Supporting Information Text S1.

### 2.2 Population Structure and Genetic Diversity

To investigate broad‐scale population structure across sampling localities, we used a standard PCA approach in PLINK (Purcell et al. 2007) and a maximum likelihood approach in Admixture (Alexander et al. 2009), where a range of population clusters (*k*) was defined between 2 (to account for the two known species, *H. mitchelli* and *H. rubrovermiculatus* ) and 10 (a reasonable maximum number of clusters based on mtDNA research, Barratt, Bwong, et al. 2017; Bwong et al. 2020). We determined the most likely number of clusters represented by our data using the 10‐fold cross validation (CV) procedure. To complement the admixture analysis, we also used a sparse non‐negative matrix factorization approach (sNMF R package, Frichot et al. 2014) and a Bayesian approach (fastStructure, Raj et al. 2014) with the same range of *k* (2–10) evaluated using the cross‐entropy approach of 10 replicates for each iteration. We generated genetic diversity metrics per sampling locality (nucleotide diversity *π*, inbreeding coefficient F_IS_, observed (*H*_o_) and expected heterozygosity *H*_e_) based on an 80% complete SNP data matrix using the *‐‐fstats* option in Stacks for all sites (i.e., fixed and variant) and for variant sites only. Furthermore, we approximated effective population size (*N*_e_) by calculating the site frequency spectrum (SFS) from Stacks output files (without a minor allele filter, to maximize representation of rare alleles) using easySFS (Overcast 2021) and then running this in *momi2* (MOran Models for Inference, Kamm et al. 2019). We calculated *N*_e_ as the mean of 100 repetitions using the *momi.model.get.params()* function for each of the major clusters identified from the population structure analyses, as well as for within‐cluster population substructure and per sampling locality.

### 2.3 Landscape Barriers and Genetic Differentiation

We used EEMS (Estimated Effective Migration Surfaces) v.0.0.0.9000 (Petkova et al. 2016) to visualize potential landscape barriers and connectivity between sampling localities based on genetic dissimilarity. The method is purely based on the genetic data and GPS coordinates of samples and highlights geographic areas where genetic similarity is higher (i.e., a signal of increased gene flow) or lower (i.e., geographic barriers) than expected under isolation‐by‐distance using spatial and SNP data. Using a PLINK (Purcell et al. 2007) format input file (generated using the *populations* module in Stacks), we set the number of cells in the modeling area (termed ‘demes’ in EEMS) to 500 based on the size of our sampling area and the number of demes representing realistic units required to fill that habitat. This data set was then run with the SNP version of EEMS (*runeems snps*). We used an MCMC length of 1,000,000 with a burn‐in of 100,000 for each of three replicates and verified that the MCMC chains had converged. We combined results using the EEMS R plotting (*rEEMSplot*) package and plotted surfaces of effective migration and diversity rates separately. To aid interpretation of the EEMS analyses, we calculated pairwise genetic differentiation (*F*_ST_) using the *diffCalc* function of the diveRsity R package (Keenan et al. 2013) with sample sizes corrected following Weir and Cockerham (1984), at an alpha significance level of 0.05 across 100 bootstrap replicates.

### 2.4 Species Distribution Modelling

To predict the habitat suitability for *H. mitchelli* and *H. rubrovermiculatus* across their geographic ranges, we used species distribution models (SDMs) for present environmental conditions as well as three different shared socioeconomic pathways (SSP) scenarios (SSP1—‘Sustainability’, SSP2—‘Middle of the road’, and SSP5—‘Fossil‐fuelled development’) and three future time periods (2021–2040, 2041–2060, and 2061–2080). This enables direct comparisons of the species' current habitat suitability with a total of nine future climate change scenarios that incorporate both climate and land use change scenarios using integrated assessment models (IAMs).

To prepare presence data for our SDMs, we integrated our georeferenced data with occurrence records from Barratt, Bwong, et al. (2017) and Bwong et al. (2020), along with data downloaded from the Global Biodiversity Information Facility and iNaturalist (search terms; species = ‘ *Hyperolius mitchelli* ’ OR ‘ *Hyperolius rubrovermiculatus* ’, GBIF 2024), recorded no earlier than 2009 to match the temporal timescale of our genomic data and to give an accurate depiction of the species' current ranges. We cleaned our occurrence data from GBIF and iNaturalist by using the R package ‘CoordinateCleaner’ (Zizka et al. 2019) to detect any spatial outliers, transversed coordinates, country centroids, or biodiversity institutes (e.g., museums, zoos, etc.). We then spatially rarefied our presence data to account for spatial autocorrelation (retaining only presence points within a geographic buffer of 10 km using the *spThin* R package (Aiello‐Lammens et al. 2015)). Pseudoabsence (background) points (*n* = 10,000) were selected from a buffer of 2 degrees around presence points to capture potential suitable environmental conditions for the species where it has not yet been recorded. The buffer also excluded a radius of 0.1 degrees around each presence point to avoid selecting background points within an area where the same population may be present. We downloaded temperature and precipitation‐related bioclim variables 1–19 from the Worldclim 2 data set (Fick and Hijmans 2017) and prepared a raster representing slope (i.e., topographic heterogeneity), which was calculated using a digital elevation model based on SRTM data (also available at the Worldclim2, https://www.worldclim.org/), and a map of land cover (Schipper et al. 2020). Land cover was re‐categorized into nine classes following Razgour et al. (2019) to reduce complexity in the models. Before building SDMs, we used the *vif()* function of the *usdm* R package (Naimi 2012) to exclude variables with high Variance Inflation Factors that were highly collinear (VIF > 5) with one another. From the initial 21 predictor variables, 11 were found to have a high collinearity. We thus retained 10 predictors for SDMs, which represented temperature and precipitation‐related variables, as well as slope and land cover. Predictor spatial resolution across all time periods was 30 arc sec (~1 km^2^ pixels). We used the *biomod2* R package (Thuiller et al. 2009, 2024) to build SDMs firstly using five iterations of three algorithms (Random Forest, Generalized Additive Models, and Maxent), which were then evaluated with the *get_evaluations()* function based on the balance of true vs. false positives represented by their receiver operating characteristic (ROC) curve. Models that passed our threshold of a ROC metric > 0.75 were carried forward to build a final ensemble SDM, weighted by the ROC of each individual model, where we summarized variable importances across all model runs. To evaluate predicted loss and gain of suitable habitat in the future, we performed a quantitative analysis using raster algebra for the SDMs between the present and each of the nine modelled scenarios (SSP1, SSP2, and SSP5 across each of the three time periods). Quantitative calculations of habitat suitability change were summarized in a 10 km buffer around each population between time periods. We represented habitat suitability change for each population compared to the current baseline model in terms of ‘loss’ (i.e., a decrease in habitat suitability), being ‘stable’ (i.e., no change), or ‘gain’ (i.e., an increase in habitat suitability).

### 2.5 Assessing Local Adaptation and Calculating Genomic Offset per Population

Candidate SNPs putatively involved in local adaptation were identified using redundancy analysis (RDA, Oksanen et al. 2012), based on recommended best practices for the *rda* function (Capblancq and Forester 2021). To conduct our analyses, we extracted environmental data relevant to each sampling locality based on their geographic coordinates and selected four uncorrelated climate variables (VIF < 5) that showed the highest variable importances based on SDM outputs (bioclim 14—Precipitation of Driest Month, bioclim 7—Temperature Annual Range, bioclim 12—Annual Precipitation, and bioclim 19—Precipitation of Coldest Quarter). As well as their importance in explaining model variance in the SDMs, we regard these predictors as particularly ecologically relevant to represent climatic extremes faced by the *H. mitchelli* complex in East Africa during the dry season. This is typically between June and September but has more recently extended into the rainy seasons due to climate change (Funk et al. 2023). We followed Razgour et al. (2019) to optimize our analyses, identifying putatively adaptive SNPs with a standard deviation of > 3 from the mean RDA loadings. Although we could have been less stringent in our criteria for categorizing putatively adaptive SNPs, we opted to be more conservative by using a threshold of three standard deviations from the mean loadings to categorize adaptive SNPs (two‐tailed *p*‐value = 0.0027), thus attempting to minimize false positive rates at the expense of missing out on true positives (Capblancq and Forester 2021). We then used the approach of Barratt, Onstein, et al. (2024) to categorize individuals using only putatively adaptive SNPs (i.e., by plotting the position of all individuals in the RDA space relative to the biplot arrows of each of the four predictors). We mapped the proportions of individuals in each sampling locality that fall in defined adaptive categories using the ‘mapPies’ function of the *rworldmap* R package (South 2011), providing an overview of the degree of putative local adaptation at each sampling locality across the four predictors.

Based on the identified putatively adaptive SNPs and the geography of sampling localities, we quantified genomic offset per sampling locality, a metric reflecting potential vulnerability to predicted global change through a disruption of genotype–environment associations. To do this, we used a machine learning approach that detects non‐linear relationships between genotypes and local environmental conditions. We estimated current allelic frequency–climate relationships for putatively adaptive SNPs using the approach developed by Fitzpatrick et al. (2021), implemented in the *gradientForest* R package (Ellis et al. 2012). Genomic offsets were then computed as Euclidean distances, representing the genomic mismatch between current and future climatic conditions based on the gradient forest model predictions (Fitzpatrick and Keller 2015). Present environmental conditions (over the 1981–2010 period) were extracted per sampling locality from each climatic raster using the *terra* R package (Hijmans 2020), considering bioclimatic variables (Worldclim2). Genomic offsets were calculated using predictions from the gradient forest model and raster values representing future climate conditions projected for each of the scenarios used for the SDMs (three time periods: 2021–2040, 2041–2060, and 2061–2080; and three shared socioeconomic pathways: SSP1, SSP2, SSP5).

## 3 Results

### 3.1 Genomic Data Processing and SNP Filtering

After demultiplexing and filtering our raw Illumina sequences, we retained a total of 186.29 million reads (mean per sample = 3.39 million, range = 1.59–6.78 million). With our optimized Stacks parameters of *m* = 5, *M* = 2, and retaining only SNPs present in 80% of all individuals processed, we generated a final data set of 3849 unlinked SNPs genotyped in our *H. mitchelli* complex samples (*n* = 55).

### 3.2 Population Structure and Genetic Diversity

The PCA revealed three main population clusters for the samples from Kenya, Malawi, and Tanzania, with the Tanzanian cluster demonstrating some geographical population substructure. Localities 8 (Kimboza) and 19 (Kiwengoma) were located in a ‘grey zone’ (marked by a grey ellipse) between the Northern, Central, and Southern Tanzanian clusters (Figure 1A). Admixture, fastStructure, and sNMF analyses were congruent that the population structure exhibited by our sampling is between *k* = 2 and *k* = 4 based on cross‐validation (CV), log likelihoods, and cross‐entropy (CE), respectively, and the same geographic population substructure from the PCA is visible at higher values of k across all three analyses (see Figures 1B and S2). Due to the general congruence in population structure between the PCA, Admixture, and fastStructure with the sNMF analyses, we conclude that three broad population clusters are a reasonable representation of the underlying sampling. We further tested the validity of population clustering from *k* = 2 to 5, as suggested by our population structure analyses using AMOVA in the poppr R package (Kamvar et al. 2014, 2015), which also supported three population clusters as the most likely explanation for the data (see Φ_ST = 0.339 at *k* = 3, Table S2). However, given the clearly defined geographical population substructure in the Tanzanian cluster, we believe that it likely represents a large metapopulation.

Geographically, the analyses showed that population structure across iterations of *k* often coincides with potential riverine (Rufiji, Kilombero, Great Ruaha, and Wami rivers) and montane barriers (Eastern Arc mountains), largely matching the structure defined in a previous phylogeographic study (Bwong et al. 2020) except in terms of subdivision of the Tanzanian cluster. Across population clusters, there was some degree of shared co‐ancestry or gene flow, particularly in sNMF analyses, suggesting potential hybrid zones with admixture between identified clusters and lower admixture for isolated peripheral sampling localities (Figure 1C). Pare (18) and Kiwengoma (19) in Tanzania, as well as Luwawa (20) in Malawi, shared substantial co‐ancestry or gene flow with all four clusters based on sNMF analyses and often split into separate clusters at higher values of *k* (Figure S2). In terms of standing neutral genetic diversity, both nucleotide diversity and observed heterozygosity were generally lowest (*π* = 0–0.05, *H*_o_ = 0.0005–0.001) in Malawi (20) and Pare (18), average (*π* = 0.05–0.1, *H*_o_ = 0.001–0.002) in Kenya (1–4), and highest (*π* > 0.1, *H*_o_ = 0.002–0.003) throughout the remainder of Tanzania (5–19) (Figure 1D). Detailed per‐locality estimates of *H*_o_, *H*_e_, *F*_IS_, and *π* can be found in Table S3. Effective population size (*N*_e_), calculated in *momi2* based on population‐level site frequency spectra, supported the genetic diversity analyses, with larger and more genetically diverse population clusters having higher *N*_e_ (Table S4). Relatively, the Tanzanian cluster suggests a much larger effective population size (*N*_e_ = 5053) than the Kenya (*N*_e_ = 1479) and the Malawi cluster (*N*_e_ = 620), and Northern and Central Tanzania suggest higher *N*_e_ than Southern Tanzania (*N*_e_ = 1727, 2237, and 826, respectively). Per locality estimates of *N*_e_ are much smaller, ranging from 72 (Kivumoni in Kenya) to 492 (Kimboza in Tanzania), with a mean *N*_e_ of 258.

### 3.3 Landscape Barriers and Genetic Differentiation

**FIGURE 2:**
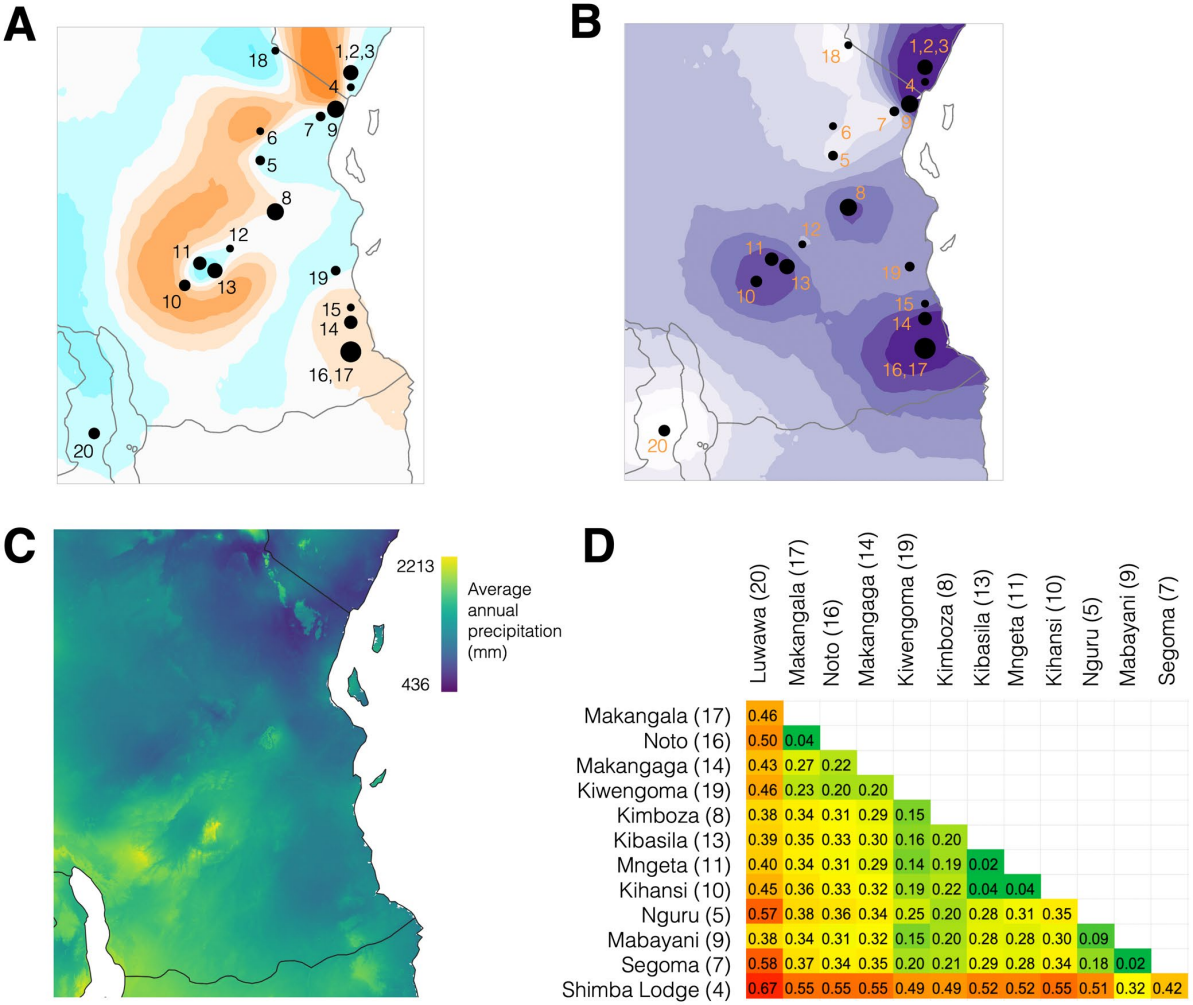
Estimated effective migration and diversity surfaces across *H. mitchelli* and *H. rubrovermiculatus* sampling localities (black dots). (A) Blue represents areas of higher than predicted migration (i.e., connectivity), orange represents areas of lower than predicted migration (i.e., barriers). (B) Darker purple areas represent areas of higher effective diversity. (C) Rainfall map (annual precipitation in mm, downloaded from Worldclim2, Fick and Hijmans 2017) for the study region, dark blue colors are areas of lower precipitation, light green/yellow colors are areas of higher precipitation. (D) Genetic differentiation (*F*_ST_) across sampling localities with at least two individuals was calculated using the sample size corrected approach of Weir and Cockerham (1984).

Landscape barriers analysis in EEMS supported population structure analyses and suggested that although most coastal and lowland sampling localities are potentially well connected (high effective migration, blue in Figure 2A), several barriers (low effective migration, orange in Figure 2A) coinciding with areas west of Eastern Arc mountain blocks with lower rainfall levels (see darker blue regions in Figure 2C) exist that potentially hinder gene flow. Effective diversity surfaces calculated in EEMS (Figure 2B) also suggested that hotspots of higher effective diversity mainly coincided with those reflected by higher observed heterozygosity (Kenyan (1–4) and Tanzanian (7, 8, 9, 10–13, 14–17)). Genetic differentiation (*F*_ST_) ranged from 0.02 to 0.67 (mean = 0.32), with Malawi (0.38–0.67, mean = 0.47) and Kenya (0.32–0.67, mean = 0.51) being the most differentiated from the remaining sampling localities. Sampling localities near the Udzungwa Mountains in Tanzania (10, 11, 13) showed a lower variance in *F*_ST_ (0.02–0.38, mean = 0.25) with the exception of Kihansi (10) (0.04–0.55, mean = 0.29) and those in southern Tanzania Makangaga (14), Noto (16), and Makangala (17) (0.04–0.55, mean = 0.32, Figure 2D).

### 3.4 Species Distribution Modelling

**FIGURE 3:**
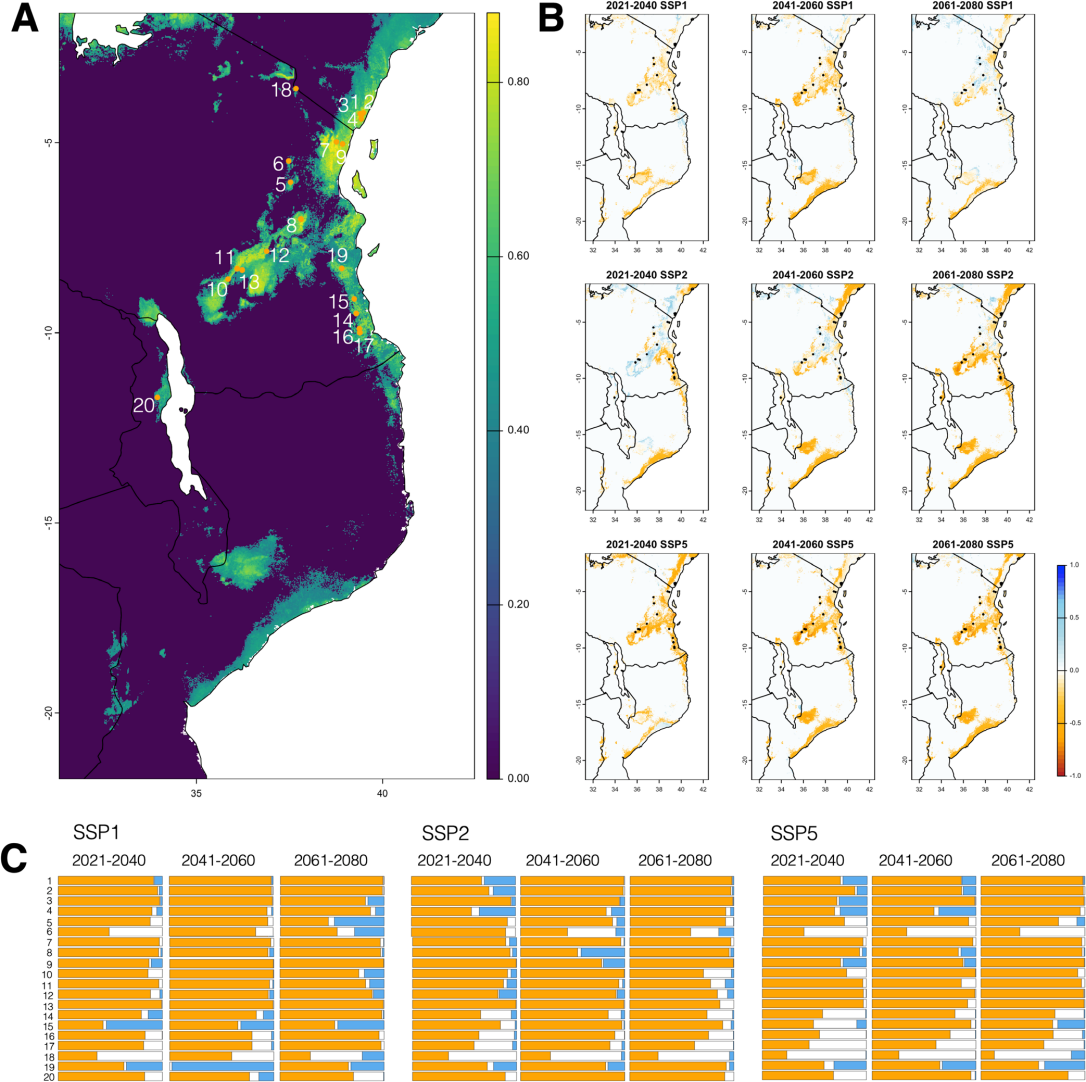
Predicted habitat suitability change over time for the *H. mitchelli* complex. Sampling locality numbers labelled (1–20). (A) Current ensemble SDM (scale bar denotes habitat suitability from 0 to 1). (B) Predicted habitat suitability loss (orange) and gain (blue) based on ensemble model SDM predictions. For each time period and SSP (Shared Socioeconomic Pathway), the habitat suitability change is represented, relative to the present SDM prediction (continuous scale, −1 = total loss of habitat suitability, 0 = no change, 1 = gain of new suitable habitat). (C) Summaries of proportional habitat suitability loss (orange), gain (blue), and no change (white) per sampling locality calculated in a 10 km^2^ buffer around the sampling localities. SDM output maps for each time period can be found in Figure S4.

From an initial 307 presence records (55 from our genomic samples and 252 from GBIF, iNaturalist, and Bwong et al. 2020), we discarded five records flagged by CoordinateCleaner (Zizka et al. 2019) as country centroids or museum/biodiversity institutes, as well as those far outside (> 250 km) of the known range. We retained a total of 44 unique presence records after controlling for spatial autocorrelation (spatial thinning within a radius of 10 km). These were then used as presence points for SDMs. Our study uses a relatively modest number of presence records, but these fall within the range where previous work has shown that reliable SDMs can still be generated (Pearson et al. 2007; Van Proosdij et al. 2015). The predictive power of our models is further strengthened by the ensemble framework, which integrates multiple algorithms to reduce individual biases and improve robustness (Breiner et al. 2015; Araújo et al. 2019). Combined with the spatial scale and ecological relevance of the study area, this approach ensures that our models provide meaningful and reliable predictions despite modest sample sizes. Species Distribution Models ranked bioclim 7 (Temperature annual range), bioclim 12 (Annual precipitation), bioclim 14 (Precipitation of driest month), and bioclim 19 (Precipitation of coldest quarter) as the predictors with the mean highest variable importance across all SDM model runs (Figure S3). The SDM for the current time period (Figure 3A) demonstrated large areas of high suitability (probability > 0.75) surrounding the known presences of *H. mitchelli* and *H. rubrovermiculatus* , encompassing the coastal regions of Kenya and Tanzania as well as the known mountain blocks of the Eastern Afromontane biodiversity hotspot (i.e., the Eastern Arc Mountains) where the species have been recorded (Pare, Usambara, Nguu, Nguru, Uluguru, and Udzungwa). Isolated areas of high habitat suitability were also predicted around the edges of Lake Malawi and Lake Victoria, the Mulanje and Mabu massifs in Mozambique, as well as areas of coastal forest around Cabo Delgado and Zambezia provinces in central Mozambique.

Future predictions of habitat suitability estimated that although much of the core distribution of the *H. mitchelli* species complex will remain mostly unchanged under future scenarios up to the year 2061–2080 based on SSP1 (‘sustainability’), increased range contraction and reduction in habitat suitability are predicted for SSP2 and SSP5 during all future time periods. For example, under SSP5 (‘Fossil‐fueled development’), most suitable habitats in Malawi and Mozambique will have almost entirely disappeared, and all remaining suitable habitat will be highly fragmented, with the contiguous distribution of the species likely broken in many coastal and lowland regions where temperature and aridity extremes are predicted to be highest (Figures 2 and S4). We summarize these changes in Figure 3.

### 3.5 Candidate SNPs, Local Adaptation, and Genomic Offset

**FIGURE 4:**
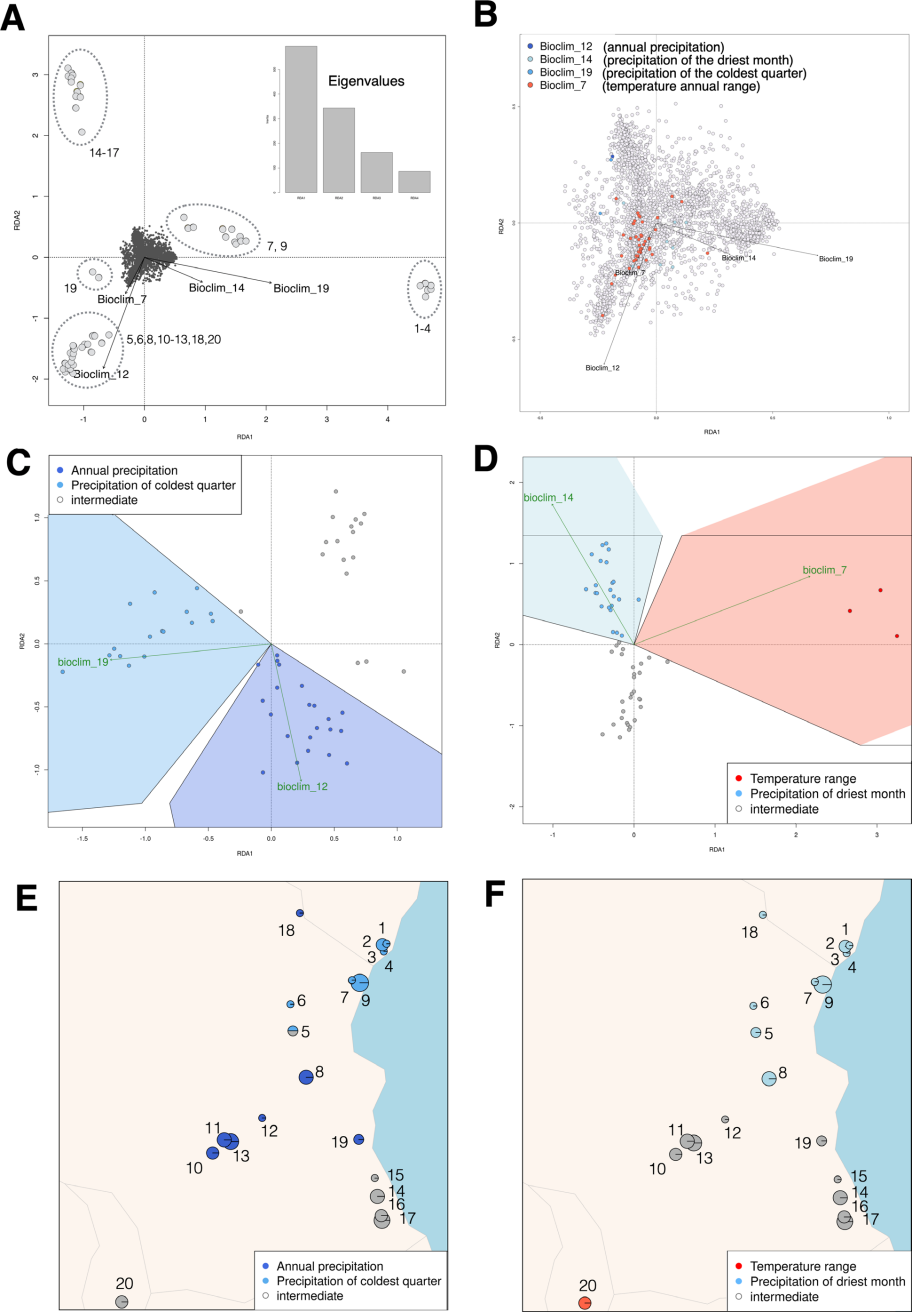
Summaries of local adaptation analyses. (A) Individual samples plotted in RDA space relative to environmental predictors (Eigenvalues for RDA are inset), with designations of sampling localities matching (Figure 1). (B) Candidate SNPs identified with genotype–environment associations, identified with RDA. SNP correlations with environmental predictors are colored according to legend. (C) Individual categorization of local adaptations to bioclim 12 and bioclim 19, and (D) Individual categorization of local adaptations to bioclim 7 and bioclim 14. (E and F) Mapped distributions of locally adapted individuals across sampling localities.

Genotype–environment association analysis performed using redundancy analysis (RDA) identified 90 genome‐wide SNPs with putative signals of local adaptation to the tested climatic variables related to precipitation and temperature (bioclim 7, bioclim 12, bioclim 14, and bioclim 19). Plotting the sampled individuals relative to the environmental data revealed that populations mostly occupy unique environmental space (Figure 4A). Considering candidate SNPs involved in local adaptation, most were temperature‐related (bioclim 7, *n* = 77), and the remainder were precipitation‐related (9 to bioclim 14, 3 to bioclim 19, and 1 to bioclim 12, Figure 4B).

Categorizing individuals based on the allele frequencies of putatively adaptive SNPs from the RDA analysis suggests spatially structured local adaptation across geographic space. Although bioclim 7 showed the highest number of SNP associations involved in putative local adaptation to temperature, only three individuals were categorized as being positively associated with this predictor. A larger number of individuals were positively associated with the remaining precipitation‐related variables (22 individuals to bioclim 12, 22 individuals to bioclim 14, 16 individuals to bioclim 19, Figure 4C,D). Spatial mapping of these putatively adapted individuals as a proportion of each sampling locality indicates that all individuals associated with bioclim 7 (temperature range) are found in Luwawa (20), and that individuals adapted to bioclim 14 (precipitation of driest month) are found across all Kenyan (1–4) and Tanzanian (5–19) populations (Figure 4C,D). Similarly, putative adaptations to bioclim 19 (precipitation of the coldest quarter) are largely in the same sampling localities (with the exception of Pare (18) and Kimboza (19)). Putative adaptations to bioclim 12 (annual precipitation) include all individuals from near the Udzungwa Mountains in Central Tanzania (10–13), Uluguru Mountains (8), and Kiwengoma (19) and Pare (18). It is noteworthy that sampling localities in southern Tanzania (14–17) show no local adaptations to any of the tested variables.

**FIGURE 5:**
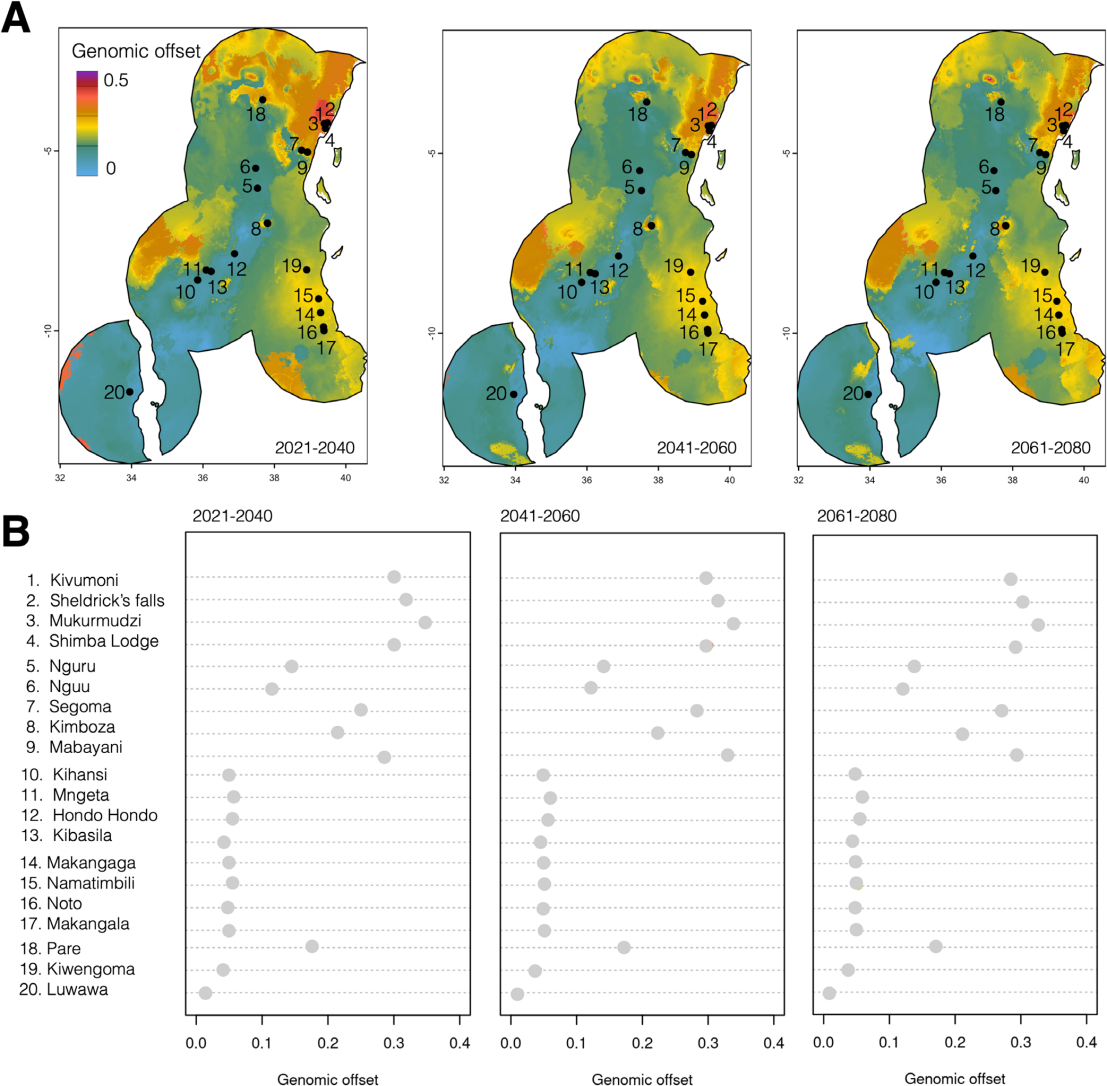
Genomic offsets for the *Hyperolius mitchelli* species complex based on allele frequency and climate change projections. (A) Maps of predicted genomic offset, clipped to a 2^o^ buffer around sampling localities. Projections for SSP2, 2021–2040, 2041–2060, and 2061–2080 are shown in the three panels. (B) Dotplots of offsets per sampling locality matching (A). Additional genomic offset maps and associated dotplots per sampling locality for all nine future scenarios can be found in Figures S5 and S6.

Modelled genomic offset between current and future environmental conditions suggested that though the majority of sampling localities, particularly in Malawi and central and southern Tanzania (5, 6, 10–17, 19, 20), will likely experience low genomic offsets, the northern part of the study region Kenya (1–4) as well as northern Tanzania (7–9, 18), will experience much higher genomic offset (Figure 5). These estimates remain consistent across all modelled SSP scenarios and future time periods (Figures S5 and S6).

## 4 Discussion

In this study, we demonstrate how multiple lines of evidence can be integrated to predict future responses of intraspecific biodiversity to human‐induced habitat fragmentation and climate change. Our spatial and genomics‐informed insights into the *Hyperolius mitchelli* species complex suggest the existence of multiple population clusters, many with idiosyncratic conservation requirements. We show that, although understanding population structure and patterns of genetic diversity is a reliable guiding basic principle for prioritizing conservation efforts, modeling climate change and using genomic indicators can provide additional powerful insights to understand the different conservation contexts and requirements across geographic space. Specifically, determining the degree of local adaptation and landscape connectivity, as well as predicting future habitat suitability and potential maladaptation using genomic offset, are important factors when considering and planning conservation management strategies. Below, we discuss how these considerations can be applied to non‐model species such as *H. mitchelli* and *H. rubrovermiculatus* , providing management recommendations for population clusters, as well as describing how the general principles of our work can be expanded to other taxonomic groups in different geographic settings with relevant modifications. In addition, we review caveats and potential limitations of our data and findings, suggesting further improvements when selecting suitable strategies to manage biodiversity using approaches with multiple lines of evidence such as ours.

### 4.1 Population Structure With Gene Flow Highlights Ongoing Low‐Level Genetic Exchange

**FIGURE 6:**
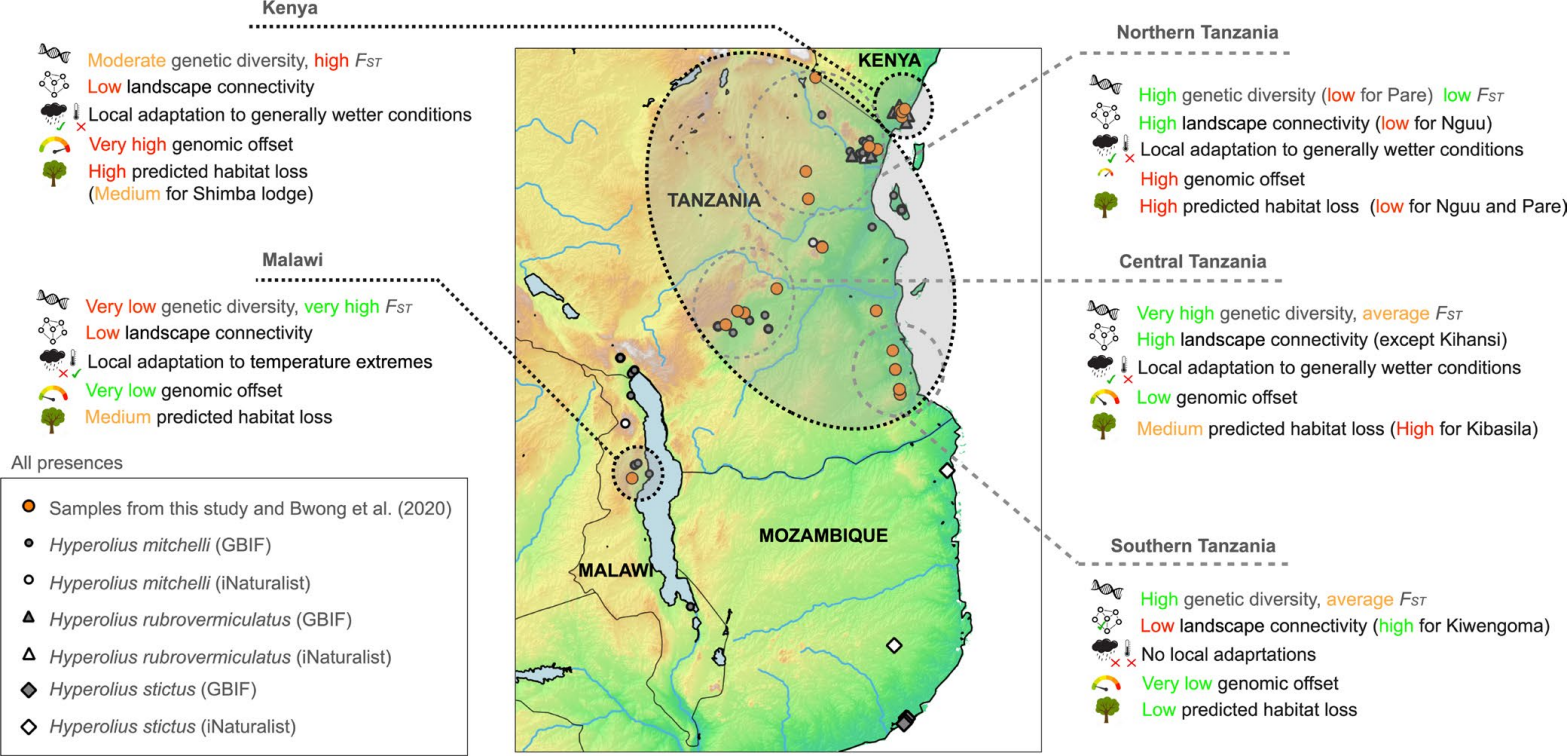
Population cluster characteristics for the *H. mitchelli* complex based on estimates of population structure, genetic diversity and differentiation, modelled landscape connectivity, local adaptations, genomic offsets, and SDM predictions. Three main population clusters (*k* = 3—Kenya, Malawi, Tanzania) were identified consistently across all population structure analyses, with substructure potentially representing three geographically separated localities in Tanzania (also visualized described on the right side of the plot, but not statistically supported by AMOVA, see Table S2). Note that localities 8 (Kimboza) and 19 (Kiwengoma) do not fall in any of the defined clusters due to their unclear ‘grey zone’ status between northern, central and southern Tanzanian population clusters. For each cluster, the results of the analyses are summarized for ease of interpretation. All known presence records collected from iNaturalist (2024) and GBIF (2024) for *H. mitchelli, H. rubrovermiculatus*, and the congeneric *H. stictus* are plotted to represent additional known occurrences that are not covered by our genomic sampling.

Based on our combined analyses and sampling, we recommend that the three discrete clusters (‘Kenya,’ ‘Tanzania,’ and ‘Malawi’) across the range of the *H. mitchelli* species complex should be maintained as separate management units (Figure 6). For the purpose of conservation, these genomic clusters should be considered as the coarsest scale, but low levels of shared ancestry or gene flow should be acknowledged as part of the population substructuring. Our results are in contrast to previous studies based on mitochondrial data (Barratt, Bwong, et al. 2017; Portik et al. 2019; Bwong et al. 2020), which showed a cluster of *H. rubrovermiculatus* and northern Tanzanian *H. mitchelli* , and a central Tanzanian and Malawian joint cluster. This discrepancy appears to stem from gene flow between clusters (Figure 1B), resulting in admixture between *H. rubrovermiculatus* and northern Tanzanian localities of *H. mitchelli* (red) and between central Tanzania and Malawi *H. mitchelli* (grey) seen primarily in monophyletic mitochondrial clusters that are not present in our genomic assessment. One possible explanation is sex‐biased dispersal: the stronger signal of gene flow in the matrilineally inherited mitochondrial data set (Bwong et al. 2020) compared to our nuclear data set suggests that females may exhibit different dispersal patterns than males, creating divergent connectivity signals. A similar result was seen in the congeneric and co‐distributed *Hyperolius substriatus* system (Lawson et al. 2025). These areas of potential gene flow occur along corridors of suitable habitat for the Kenyan and northern Tanzanian clusters, but span a significant environmental gap (Makambako Gap) between the Udzungwa Mountains in central Tanzania, which impacts a wide variety of vertebrates (Burgess et al. 2007).

Our genomic‐based results suggest that the Tanzanian cluster is potentially a large metapopulation with three subclusters also exhibiting gene flow (‘Northern Tanzania’, ‘Central Tanzania’, and ‘Southern Tanzania’). However, locations that lie in between the main three ‘Northern’, ‘Central’, and ‘Southern’ clusters (i.e., localities 8 and 19) may constitute a fourth genetic cluster or represent a hybrid zone. To understand this population substructure in more detail and ensure that all clusters and populations are characterized will require denser sampling of localities, especially around the peripheral areas of sampling in this work.

### 4.2 Management Recommendations for the *H. mitchelli* Species Complex

From a conservation perspective, improving and increasing suitable habitats and their connectivity would assist the establishment of healthier and more resilient metapopulations for the *H. mitchelli* species complex (Moor et al. 2024). This would counter the effects of forest habitat fragmentation, genetic drift, and low genetic diversity (a proxy for smaller effective population sizes) generally driven by human activities and potentially exacerbated by frequent droughts in East Africa over the past decades (Funk et al. 2023). In the northernmost parts of the *H. mitchelli* species complex range, Kenyan populations (1–4, i.e., *H. rubrovermiculatus* ) were genetically distinct, with limited admixture from the nearest adjacent populations in Northern Tanzania (5–9, 18). *Hyperolius rubrovermiculatus* is endangered (IUCN 2023) and known only from the Shimba Hills National Reserve and surrounding coastal forest region in Kenya. Bwong et al. (2020), using only mitochondrial DNA, showed that populations of *H. mitchelli* (Least Concern, IUCN 2023) from northern Tanzania are phylogenetically clustered with *H. rubrovermiculatus* in Kenya. In our work, although habitat suitability from SDMs between the populations appears to be highly contiguous, the genomic dissimilarity and apparent barriers to gene flow suggest that they are most likely a closely related but distinct taxon. Based on this information, and despite their matching putative local adaptations to wetter conditions, similarly high genomic offset predictions, moderate to high levels of genetic diversity, and high predicted habitat loss from SDMs, promoting augmented gene flow between these two areas would not be advised. Doing so could potentially lead to hybrid incompatibility or genetic swamping where genetic diversity in recipient populations is reduced due to an excess of hybrids (Todesco et al. 2016), though this is not always the case (see Ottenburghs 2021).

Towards the central and southern parts of the *H. mitchelli* species complex range, Tanzanian localities close to the Udzungwa Mountains (10–13, ‘Central Tanzania’ cluster) and the Mozambique border (14–17, ‘Southern Tanzania’ cluster), being highly genetically diverse with low differentiation from most other localities throughout Tanzania, offer an intriguing potential source for replenishing surrounding genetically impoverished areas via augmented gene flow. Their low genomic offset and relative connectivity to other localities also lend support to this idea. Similarly, many localities in the ‘Northern Tanzania’ cluster, including Segoma (7), Mabayani (9), Nguru (6), Nguu (5), demonstrate a high degree of genetic diversity and are only moderately differentiated from the remainder of the Tanzanian localities. However, contrasting local adaptations between northern and central/southern clusters suggest that promoting augmented gene flow in some cases could compromise existing local adaptations across this climatic and floristic gradient (White 1983). Furthermore, it remains to be seen clearly which population cluster the Kimboza (8) and Kiwengoma (19) localities belong to.

In the south‐westernmost part of our sampling, our single population in Luwawa (‘Malawi’, 20) is highly differentiated with low connectivity from all other populations in line with its geographical isolation at the periphery of the *H. mitchelli* complex range. It is worth noting that this population is geographically closest to the type locality of *H. mitchelli* . However, *H. mitchelli* is known from more localities in Malawi (Bwong et al. 2020 and Figure 6) that are not captured in our genomic sampling here due to low DNA quality. Low levels of genetic diversity in Malawi, as with those in northern Tanzania (i.e., Pare), suggest lower effective population sizes in these populations, congruent with our estimates of *N*_e_ (Table S3), and they could be considered an immediate concern from a conservation perspective. Throughout Malawi, further genetic screening of additional populations using higher quality DNA could help to validate whether lower genetic diversity and *N*_e_ values in this region are a common pattern (as shown for the congeneric *H. substriatus*, Lawson 2013). In Malawi, but also in other populations and localities where genetic diversity is particularly low, initiatives to create and improve habitats in an attempt to establish new populations, as well as promoting habitat connectivity among existing populations not captured by our sampling (that may be genetically evaluated first), appear to be the most appropriate strategy to maintain viable populations.

### 4.3 Applications to New Species, Systems, and Geographic Regions

Our approach demonstrates how the different lines of evidence from molecular data, spatial distributions, and predictive models can be combined to form species‐specific management plans to maintain or enhance genetic diversity and population viability (see also Hoffmann et al. 2021; Lehnert et al. 2023; Flanagan et al. 2018). As we have demonstrated with the *H. mitchelli* species complex, ecologically sensitive, short‐lived explosive breeders with relatively large census population sizes and limited dispersal abilities are highly suitable models to investigate idiosyncratic responses of populations to environmental changes across a large geographic range. Such an approach may be applied to different biological systems and co‐occurring species when suitable data are collected to formulate complementary multi‐taxon management plans. For example, our approach would easily lend itself to investigating possible management approaches for other dispersal‐limited amphibians and reptiles of sub‐Saharan Africa with widespread sampling, cryptic diversity, and available georeferenced population genomic data sets (e.g., Portik et al. 2017; Charles et al. 2018; Jaynes et al. 2022; Barratt et al. 2018; Leaché et al. 2019; Bell et al. 2015; Reyes‐Velasco, Manthey, Bourgeois, et al. 2018; Reyes‐Velasco, Manthey, Freilich, and Boissinot 2018; Miller et al. 2024; Allen et al. 2021). However, our approach is likely unsuitable for narrowly distributed species with few viable populations, for example, the Kihansi spray toad ( *Nectophrynoides asperginis* ) in central Tanzania (Sewell et al. 2024) or small mammals highly restricted to particular habitats (Demos et al. 2015).

When adopting our approach for different taxonomic groups, it should be noted that some analytical aspects may need to be modified depending on the species' biology. For migratory species with higher dispersal capabilities, such as large mammals (e.g., Bertola et al. 2024; Garcia‐Erill et al. 2022; Garcia‐Erill et al. 2024; Quinn et al. 2023; Pečnerová et al. 2021; Pedersen et al. 2018; Liu et al. 2024), population structure and signals of local adaptation are likely to be weaker than those we found in the *H. mitchelli* complex. In cases such as this, genetic differentiation and local adaptation may be low, and information at higher spatio‐temporal resolution about the extent and connectivity of suitable habitat patches (e.g., using landscape genetic approaches) could be more relevant to understand predictions of connectivity and barriers between populations through time and space and how this could feed into conservation strategies (e.g., McRae 2004; Antharaman and Manel 2021; McGarigal and Marks 1995; McGuire et al. 2016). Insights into how anthropogenic impacts and landscape use changes are impeding potential gene flow and the potential for evolutionary rescue to occur may be particularly useful to account for. This may be especially relevant for longer‐lived species that are unable to adapt to rapidly changing environmental conditions and are therefore much more reliant on dispersal as a means to counteract the negative impacts of global change (Albaladejo‐Robles et al. 2022). At the same time, with higher resolution spatial modeling approaches, the feasibility and practicality of habitat creation and restoration initiatives need to be carefully considered.

### 4.4 Limitations, Caveats, and Routes Forward

We present here a comprehensive approach to help formulate management plans for conservation units within species and species complexes; however, there are some limitations and caveats from our data, analyses, and results. First, for the *H. mitchelli* species complex, the numbers of sampled individuals are relatively modest, with several localities represented by a single individual, and some areas remain unsampled for potential populations. Like many species with cryptic diversity, its distribution in a highly biodiverse tropical region that is logistically difficult to sample comprehensively makes complete biological inventory nearly impossible (Reddy and Dávalos 2003; Hughes et al. 2021). To overcome this challenge, we used published occurrence records for all currently known populations of the species complex to build our SDMs and to summarize information for our localities (Figure 6). In doing so, the most complete knowledge of the species' distributions can be visualized with reference to the population structure we define. Building on these georeferenced localities, obtaining new high‐quality genomic data for individuals from unsampled localities, especially those in the borders between the population structure defined in this work, will help to gain a more comprehensive overview of where the geographic limits for the defined population clusters exist. Second, we acknowledge that our sampling covers a fairly large temporal range (2009–2023, see Table S1), which could potentially incorporate shifts in the genetic composition of populations. Ideally, all samples would be from a single snapshot in time; however, this is logistically difficult to coordinate across multiple geographic locations and countries. We recommend that future work should aim to have a shorter sampling period across geographic space to minimize the risk of this bias being introduced.

Third, in our analyses we do not account for the closely related and recently described *Hyperolius stictus* from two geographically isolated populations in northern Mozambique (Conradie et al. 2018), which has been suggested as a sister to the *H. mitchelli* population from Malawi (Bwong et al. 2020) based on mitochondrial DNA (see Figure 6). Given the geographic isolation of this species compared with the rest of our sampling, and the resolution of the phylogeny with genomic data, we are confident that it does not form part of the Malawi population or Southern Tanzania cluster we have defined. However, further genomic sampling would help to comprehensively address this question.

We acknowledge that whole genome sequencing (WGS) approaches at high coverage for a higher number of individuals per population (ideally > 20) would be the gold standard for estimating nucleotide diversity, observed heterozygosity, and particularly gaining more robust *N*_e_ estimates as opposed to our ddRAD‐seq strategy (i.e., a reduced representation library approach). We urge caution in interpreting the *N*_e_ results due to our limited sample size per locality and the reduced representation of the genome in our ddRAD‐seq data, which likely underrepresents rare alleles (see Marandel et al. 2020). This method captures only a small proportion (we estimate 5%–10% based on the restriction site frequency of our selected enzymes PstI and ApekI combined with our 300–500 bp fragment size selection) of the presumably large genome of *H. mitchelli* and *H. rubrovermiculatus* (~5 Gb), as seen in the congeneric *H. riggenbachi* (https://www.genomeark.org/vgp‐all/Hyperolius_riggenbachi.html), and thus affects the completeness of allele frequencies represented by the site frequency spectrum. These limitations make it difficult to reliably report the proportion of populations with *N*_e_ > 500 for the CBD Global Biodiversity Framework prohibiting reliable estimates of *N*_e_ outside of approximate and relative interpretations. Interrogating WGS data for signatures of local adaptation would also provide deeper knowledge of the genomic regions involved in local adaptation, and if annotated reference genomes are available, the identification of functional genes that may be useful in mitigating the effects of global change (Supple and Shapiro 2018; Theissinger et al. 2023). Such reference genomes could also facilitate the quantification of deleterious mutations (i.e., genetic load, Bertorelle et al. 2022; van Oosterhout 2020) across different populations to measure fitness. Future studies with comprehensive WGS data sets will allow a significant improvement in the interpretations that can be made for the conservation of this species complex.

Finally, simulation models are an extremely powerful tool to predict the outcomes of conservation actions before a decision is taken (Hoban 2014; Haller and Messer 2017; Landguth et al. 2017). Before conservation actions are taken, these models should be used to simulate potential outcomes that can then be evaluated with follow‐up monitoring to gauge the success of conservation decisions and improve them if necessary.

## 5 Conclusion

We used a widely distributed East African reed frog species complex as a model to predict how structured populations may be idiosyncratically affected by future global changes (climate and land use). By combining genome‐wide (ddRAD‐seq) data with spatial occurrence data and predictive modeling, we characterized population structure and made conservation recommendations based on population substructure and differentiation, genetic diversity, landscape barriers and connectivity, signatures of local adaptation and genomic offset, and species distribution models. Our approach provides a framework to understand how populations within species may be unevenly affected by future global changes, feeding into the conservation decision‐making process to increase population and species resilience to global change. Our approach is generalized and can therefore be expanded with larger data sets and new analytical approaches, as well as being applied in different taxonomic and geographic contexts.

## Ethics Statement

All animals were sampled in accordance with the ethical guidelines in the respective countries.

## Conflicts of Interest

The authors declare no conflicts of interest.

## Supporting information


**Figure S1:** Stacks parameter optimization for all tested parameter combinations (*m* = 3,4,5,6,7,8,9,10, *r* = 40,60,80). Panels show number of assembled sites, polymorphic sites, percent polymorphic loci, new polymorphic sites, and total SNPs across each of the 24 parameter combinations.
**Figure S2:** Population structure multi runs from *k* = 2 to 10. Plots show individual ancestry coefficients based on (A) Admixture, (B) fastStructure, and (C) sNMF analyses, individuals are grouped by sampling locality. Broad geographic region per sample noted at the top of the first ancestry plots.
**Figure S3:** Predictor variable importances for species distribution models averaged across all model runs (models used in the final ensemble).
**Figure S4:** Ensemble Species Distribution Model outputs for all time periods (2021–2040, 2041–2060, 2061–2080) and all shared socioeconomic pathways (SSP1, SSP2, SSP5).
**Figure S5:** Genomic offset predictions (clipped to a 2^o^ buffer around sampled localities) based on gradient forest analysis for all time periods (2021–2040, 2041–2060, 2061–2080) and all shared socioeconomic pathways (SSP1, SSP2, SSP5). Blue regions represent low genomic offsets (i.e., negligible or low predicted future disruption to genotype–environment associations), red regions represent high genomic offsets (i.e., high predicted future disruptions to genotype–environment associations). Sampling localities (1–20) match Figure 1.
**Figure S6:** Dotplots of genomic offset predictions (on the *x* axis, identified in Figure 1) per sampling locality for different SSP and future projections. (A) SSP1, (B) SSP2, (C) SSP5.
**Table S1:** Sample information for all tissue samples use in this study. Collector abbreviations: LPL—Lucinda P. Lawson, JGL—Joanna G. Larson, CDB—Christopher D. Barratt, BAB—Beryl A. Bwong, JVL—John V. Lyakurwa, MM—Michele Menegon, SPL—Simon P. Loader, PKM—Patrick K. Malonza. Institutional abbreviations: FMNH—Field Museum of Natural History (Chicago, USA), MCZ—Museum of Comparative Zoology (Harvard, USA), NHM—Natural History Museum (London, UK), MUSE—Museo Delle Scienze (Trento, Italy).
**Table S2:** AMOVA (Analysis of Molecular Variance). Results of AMOVA tests for population clustering between 2 and 5 as suggested by Admixture, sNMF, fastStructure, and PCA. Different groupings of population clusters were tested with 999 randomizations of the data using the *poppr* R package (Kamvar et al. 2014, 2015), with *k* = 3 being the most likely explanation of the data.
**Table S3:** Genetic diversity estimates per population, including observed heterozygosity (*H*_o_), expected heterozygosity (*H*_e_), inbreeding coefficient (FIS), and nucleotide diversity (*π*). Metrics were calculated across *all sites* (all genotyped loci, regardless of whether they are variable or not) and *fixed sites* (loci where all individuals within a population carry the same allele).
**Table S4:** Effective population size (*N*_e_) estimates using *momi2* (Kamm et al. 2019). For each population unit (i.e., the three clusters reported in the manuscript, the three subclusters in Tanzania, and unique sampling locality) the site frequency spectrum (SFS) was calculated, downprojected to maximize the number of segregating sites, and used to estimate the effective population size (*N*_e_).

## Data Availability

We provide input files required to reproduce all results in a DRYAD data and linked Zenodo code repository (https://doi.org/10.5061/dryad.h1893200t). All demultiplexed raw sequence data is available at the European Nucleotide Archive (accession PRJEB97160).
