# Supplementary material for: Different Strokes for Different Croaks: Using an African Reed Frog Species Complex as a Model to Understand Idiosyncratic Population Requirements for Conservation Management
Source: Evol Appl. 2025 Oct 2;18(10):e70164. doi: 10.1111/eva.70164 (PMC12489751; doi:10.1111/eva.70164)
Supplement: Supplementary file 1 — Figure S1: Stacks parameter optimization for all tested parameter combinations (m = 3,4,5,6,7,8,9,10, r = 40,60,80). Panels show number of assembled sites, polymorphic sites, percent polymorphic loci, new polymorphic sites, and total SNPs across each of the 24 parameter combinations. Figure S2: Population structure multi runs from k = 2 to 10. Plots show individual ancestry coefficients based on (A) Admixture, (B) fastStructure, and (C) sNMF analyses, individuals are grouped by sampling locality. Broad geographic region per sample noted at the top of the first ancestry plots. Figure S3: Predictor variable importances for species distribution models averaged across all model runs (models used in the final ensemble). Figure S4: Ensemble Species Distribution Model outputs for all time periods (2021–2040, 2041–2060, 2061–2080) and all shared socioeconomic pathways (SSP1, SSP2, SSP5). Figure S5: Genomic offset predictions (clipped to a 2o buffer around sampled localities) based on gradient forest analysis for all time periods (2021–2040, 2041–2060, 2061–2080) and all shared socioeconomic pathways (SSP1, SSP2, SSP5). Blue regions represent low genomic offsets (i.e., negligible or low predicted future disruption to genotype–environment associations), red regions represent high genomic offsets (i.e., high predicted future disruptions to genotype–environment associations). Sampling localities (1–20) match Figure 1. Figure S6: Dotplots of genomic offset predictions (on the x axis, identified in Figure 1) per sampling locality for different SSP and future projections. (A) SSP1, (B) SSP2, (C) SSP5. Table S1: Sample information for all tissue samples use in this study. Collector abbreviations: LPL—Lucinda P. Lawson, JGL—Joanna G. Larson, CDB—Christopher D. Barratt, BAB—Beryl A. Bwong, JVL—John V. Lyakurwa, MM—Michele Menegon, SPL—Simon P. Loader, PKM—Patrick K. Malonza. Institutional abbreviations: FMNH—Field Museum of Natural History (Chicago, USA), MCZ—Museum of Comparative Zo [file EVA-18-e70164-s001.docx]

**Supplementary Information: Different strokes for different croaks: using an African reed frog species complex as a model to understand idiosyncratic population requirements for conservation management**

Christopher D. Barratt^1,2,3,4^, Beryl A. Bwong^5^, Lucinda P. Lawson^6,7^, John V. Lyakurwa^8^, Sebastian Steinfartz^2^, Hendrik Müller^1,9,11^, Robert Jehle^10^ & Simon P. Loader^11^

*^1^ German Centre for Integrative Biodiversity Research (iDiv), Leipzig, Germany*

*^2^ University of Leipzig, Leipzig, Germany*

*^3^ Naturalis Biodiversity Center, Leiden, the Netherlands*

*^4^ Animal Breeding and Genomics, Wageningen University and Research, the Netherlands*

*^5^National Museums of Kenya, Nairobi, Kenya*

*^6^ University of Cincinnati, Cincinnati, USA*

*^7^ Cincinnati Children’s Hospital and Medical Center, Cincinnati, USA*

*^8^ University of Dar es Salaam, Dar es Salaam, Tanzania*

*^9^ Central Natural Science Collections, Martin Luther University Halle-Wittenberg, Halle, Germany*

*^10^ University of Salford, Salford, UK*

*^11^ Natural History Museum, London, UK*

**Supplementary Text S1.**

To verify that there was no bias in our combined SNP dataset for both species, we performed an additional SNP discovery step on each species separately using the same pipeline but without applying a minor allele frequency (MAF) filter. This allowed us to compare the extent of species-specific vs. shared SNPs and to evaluate how much of the diversity in each species is represented in the final filtered SNP set used in our analyses (3849 SNPs, filtered at MAF > 0.05). We found a total of 34,587 SNPs identified in *H. mitchelli*-only and a total of 51,098 SNPs identified in *H. rubrovermiculatus*-only. Of these, there were 578 shared SNPs shared between the two species. When calling SNPs in both *H. mitchelli* and *H. rubrovermiculatus* together (i.e. the combined dataset), 1052 SNPs from the *H. mitchelli*-only dataset were included, and 455 *H. rubrovermiculatus*-only SNPs were retained. This demonstrates that a substantial number of SNPs are unique to each species, with limited overlap, which is expected given the genetic and geographic divergence between species. Importantly, both species retain hundreds of SNPs in the combined dataset that passed the MAF filter, despite the sample size imbalance. This suggests that neither species is systematically excluded from the final SNP set. To further verify that SNP filtering in the combined dataset did not bias our downstream results, we compared estimates of genetic diversity (π, H_e_, H_o_, F_IS_) calculated from the combined dataset against those obtained from species-specific SNP datasets (no MAF filter). The values were highly similar between datasets, providing further evidence that the combined dataset captures within-species diversity well and does not disproportionately underrepresent diversity in the species with fewer samples. In summary we conclude that the use of a combined SNP dataset, filtered at MAF > 0.05, provides a balanced representation of genomic variation across both species and does not introduce significant bias into the population genomic analyses presented in this study.

**
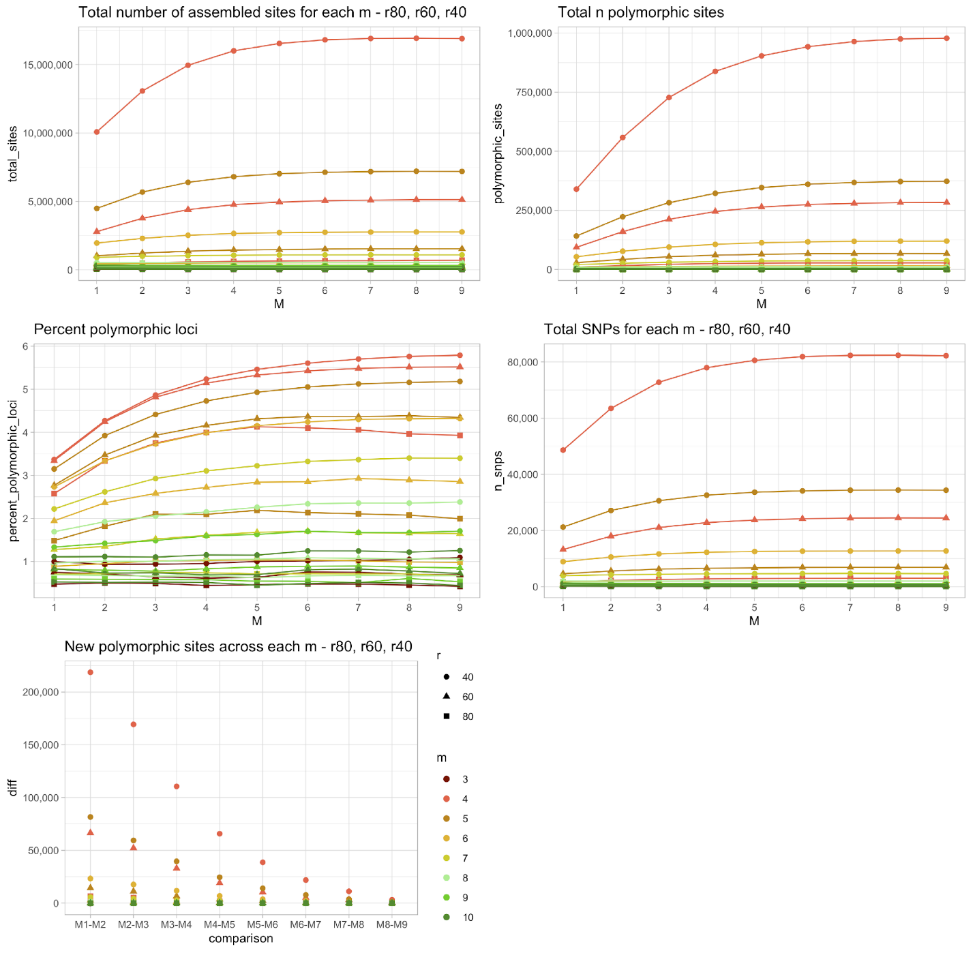
**

**Fig. S1.** Stacks parameter optimisation for all tested parameter combinations (m = 3,4,5,6,7,8,9,10, r = 40,60,80). Panels show number of assembled sites, polymorphic sites, percent polymorphic loci, new polymorphic sites and total SNPs across each of the 24 parameter combinations.


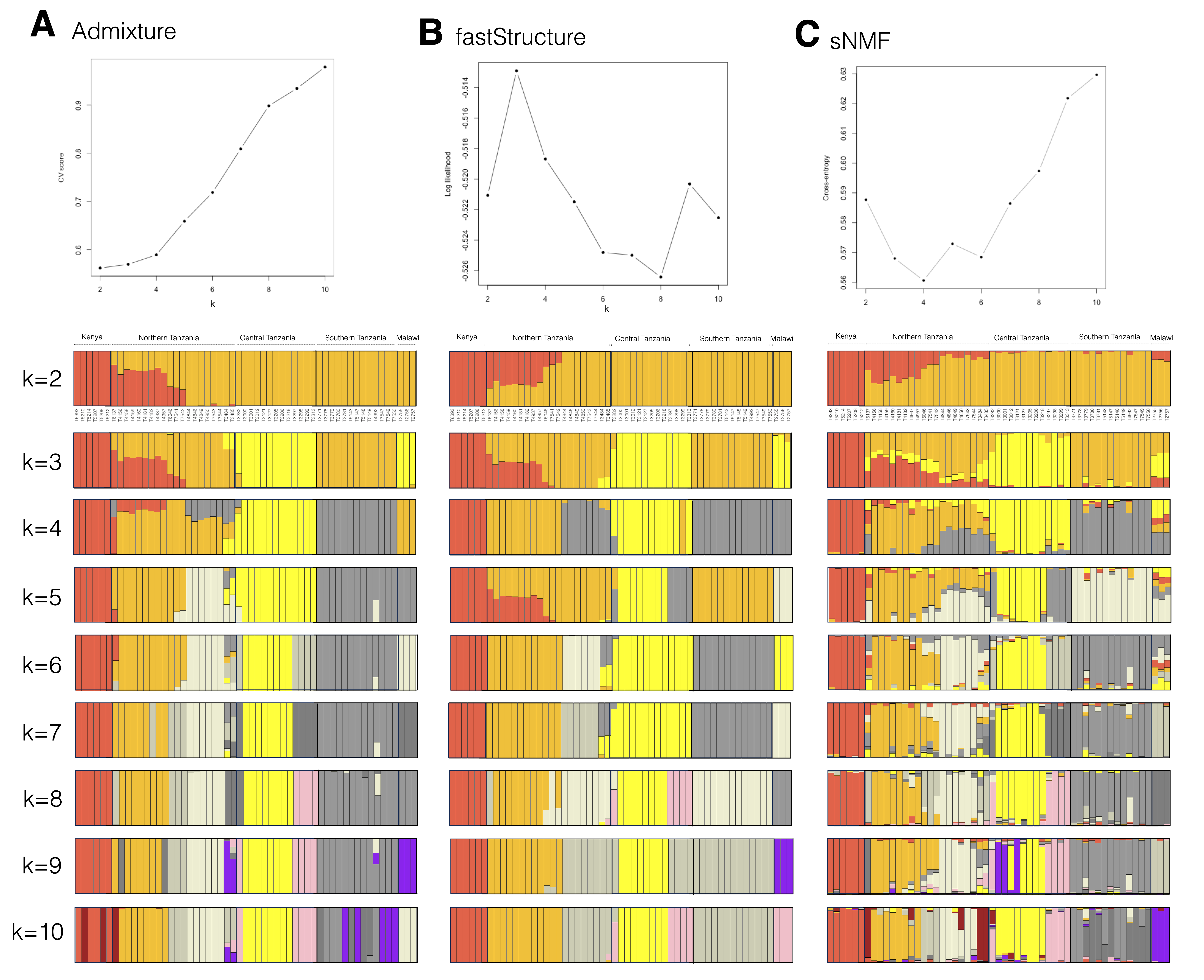


**Fig. S2.** Population structure multi runs from k=2 to 10. Plots show individual ancestry coefficients based on A) Admixture, B) fastStructure and C) sNMF analyses, individuals are grouped by sampling locality. Broad geographic region per sample noted at the top of the first ancestry plots.


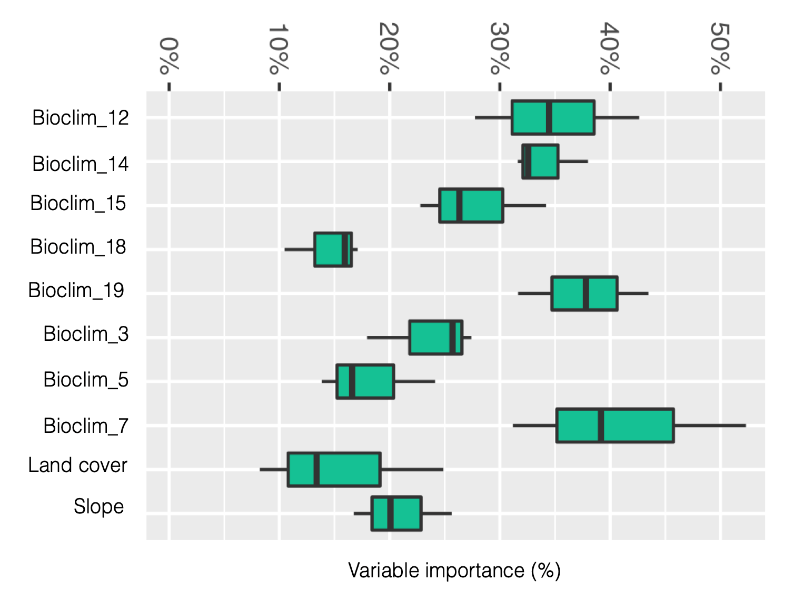


**Fig. S3.** Predictor variable importances for Species Distribution Models averaged across all model runs (models used in the final ensemble)


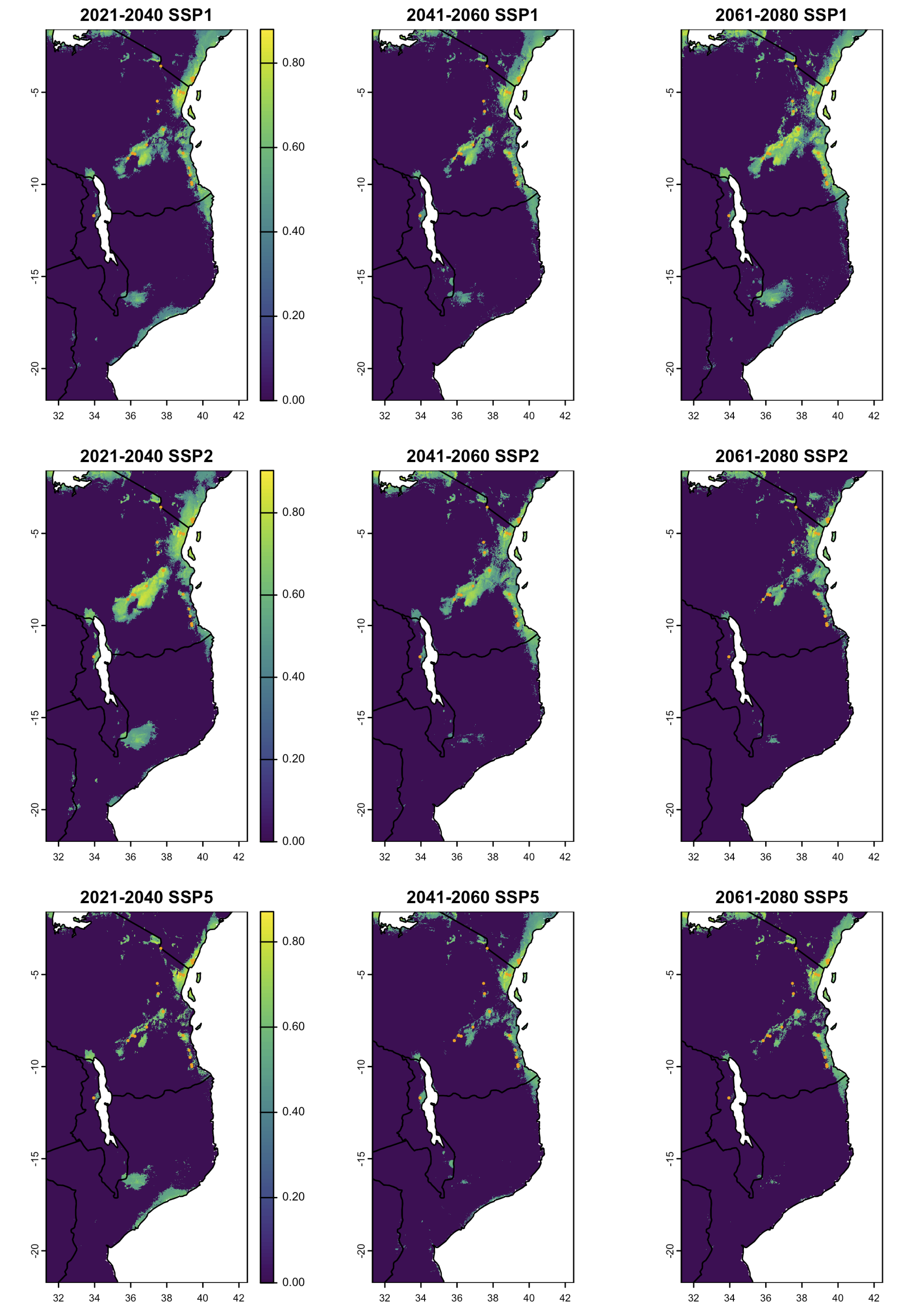
**Fig. S4.** Ensemble Species Distribution Model outputs for all time periods (2021-2040, 2041-2060, 2061-2080) and all shared socioeconomic pathways (SSP1, SSP2, SSP5).


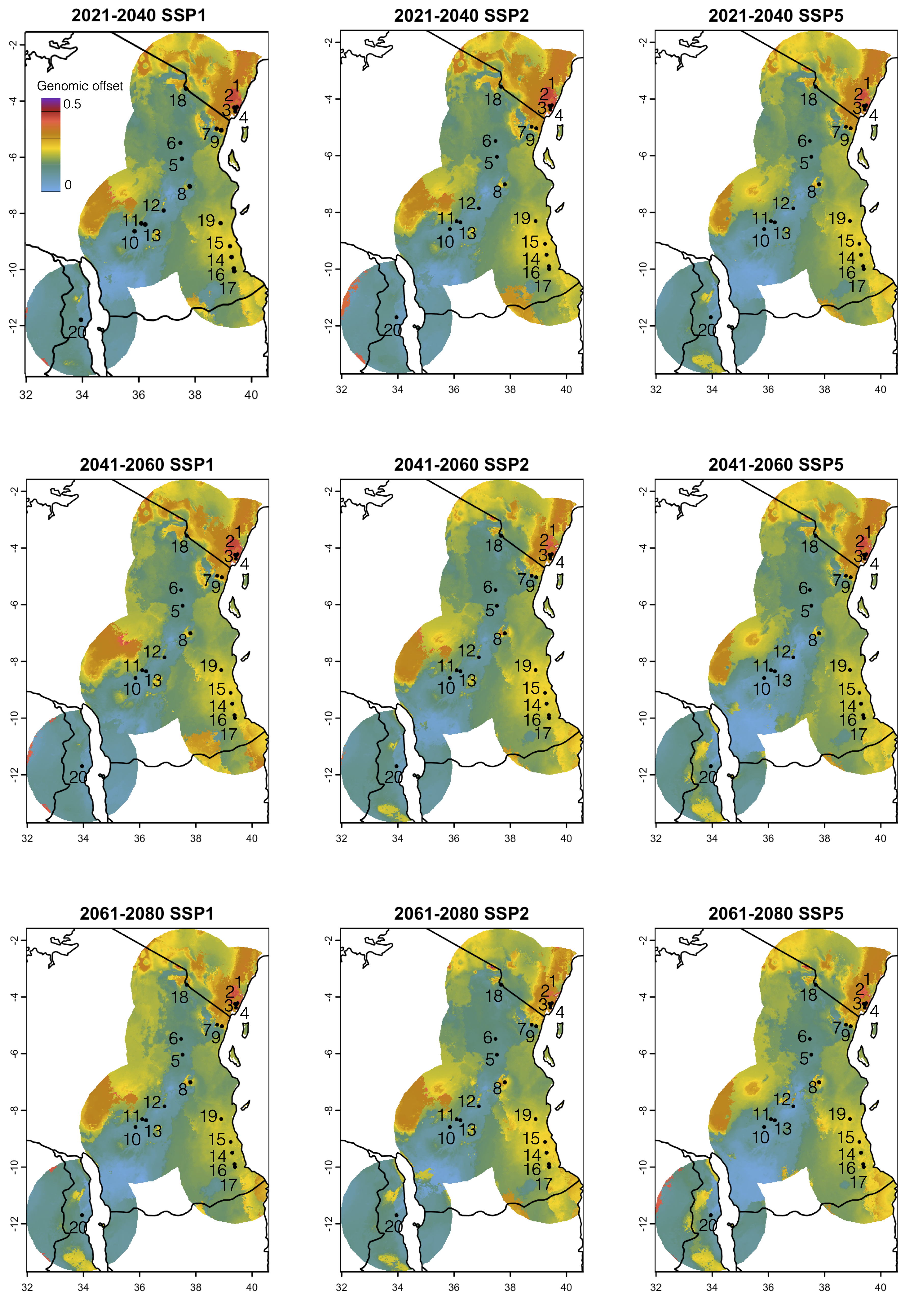
**Fig. S5.** Genomic offset predictions (clipped to a 2 degree buffer around sampled localities) based on gradient forest analysis for all time periods (2021-2040, 2041-2060, 2061-2080) and all shared socioeconomic pathways (SSP1, SSP2, SSP5). Blue regions represent low genomic offsets (i.e. negligible or low predicted future disruption to genotype-environment associations), red regions represent high genomic offsets (i.e. high predicted future disruptions to genotype-environment associations). Sampling localities (1-20) match Fig. 1.


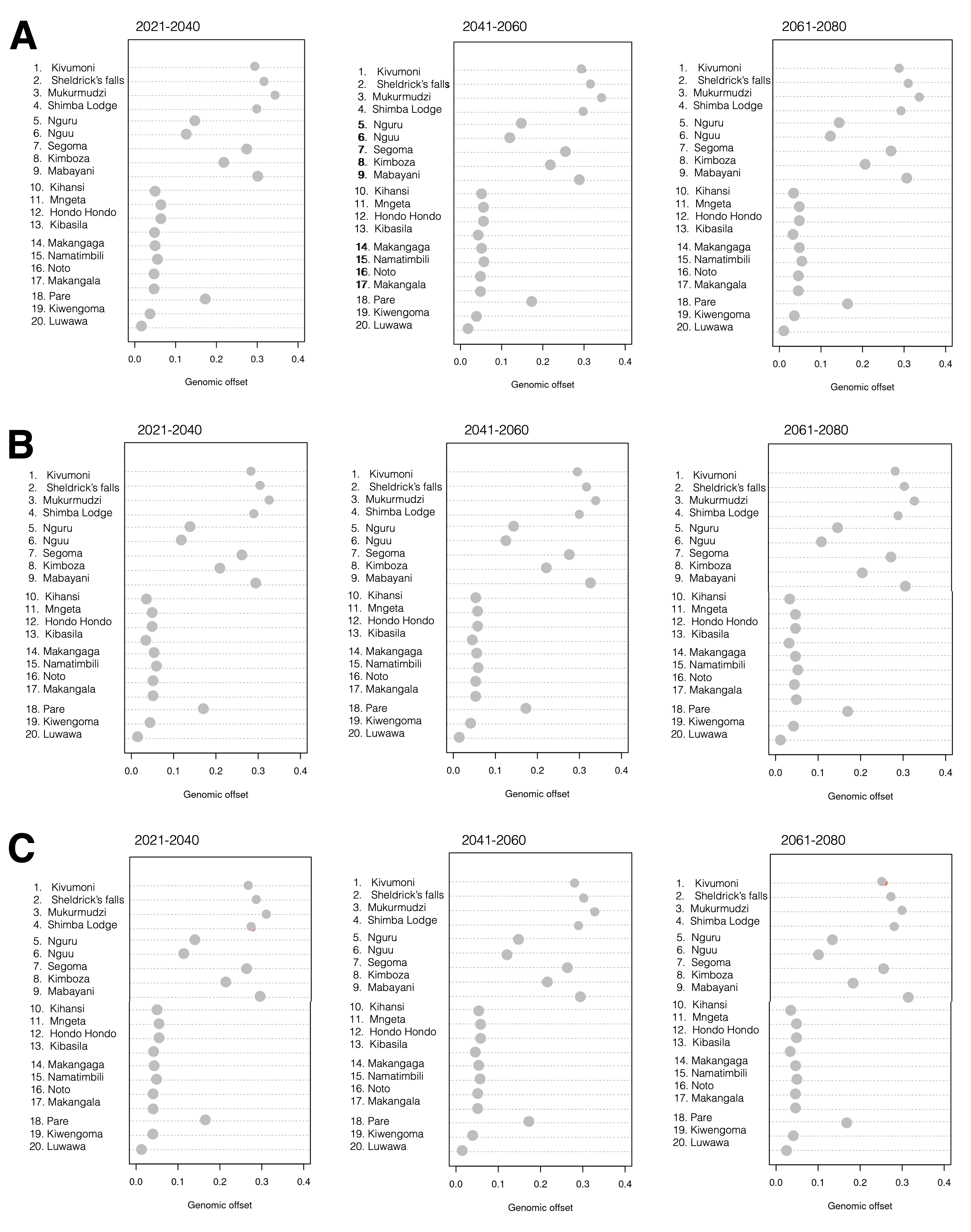


**Fig. S6.** Dotplots of genomic offset predictions (on the x axis, identified in Fig. 1) per sampling locality for different SSP and future projections. A) SSP1, B) SSP2, C) SSP5.

**Table S1.** Sample information for all tissue samples use in this study. Collector abbreviations: LPL – Lucinda P. Lawson, JGL – Joanna G. Larson, CDB – Christopher D. Barratt, BAB – Beryl A. Bwong, JVL – John V. Lyakurwa, MM – Michele Menegon, SPL – Simon P. Loader, PKM – Patrick K. Malonza. Institutional abbreviations: FMNH – Field Museum of Natural History (Chicago, USA), MCZ – Museum of Comparative Zoology (Harvard, USA), NHM – Natural History Museum (London, UK), MUSE – Museo Delle Scienze (Trento, Italy).

| Sample | Fieldtag | Species | Country | Locality | Lat | Lon | Elevation (m) | Collector | Year | Institute |
| --- | --- | --- | --- | --- | --- | --- | --- | --- | --- | --- |
| T2755 | FMNH 274980 | *Hyperolius mitchelli* | Malawi | Luwawa | -11.69809 | 33.9498 | 1287 | LPL | 2009 | FMNH |
| T2756 | FMNH 274982 | *Hyperolius mitchelli* | Malawi | Luwawa | -11.69809 | 33.9498 | 1287 | LPL | 2009 | FMNH |
| T2757 | FMNH 274985 | *Hyperolius mitchelli* | Malawi | Luwawa | -11.69809 | 33.9498 | 1287 | LPL | 2009 | FMNH |
| T4992 | MCZ A-32199 | *Hyperolius mitchelli* | Tanzania | Hondo Hondo | -7.855815 | 36.884193 |  | JGL | 2012 | MCZ |
| T3000 | CB 13.011 | *Hyperolius mitchelli* | Tanzania | Makangala | -9.99418 | 39.38803 | 254 | CDB | 2013 | NHM |
| T3001 | CB 13.012 | *Hyperolius mitchelli* | Tanzania | Makangala | -9.99418 | 39.38803 | 254 | CDB | 2013 | NHM |
| T3012 | CB 13.027 | *Hyperolius mitchelli* | Tanzania | Makangala | -9.99418 | 39.38803 | 254 | CDB | 2013 | NHM |
| T3121 | CB 13.144 | *Hyperolius mitchelli* | Tanzania | Makangala | -9.99418 | 39.38803 | 254 | CDB | 2013 | NHM |
| T3127 | CB 13.150 | *Hyperolius mitchelli* | Tanzania | Makangala | -9.99418 | 39.38803 | 254 | CDB | 2013 | NHM |
| T3205 | CB 13.228 | *Hyperolius mitchelli* | Tanzania | Noto | -9.89532 | 39.37409 | 277 | CDB | 2013 | NHM |
| T3206 | CB 13.229 | *Hyperolius mitchelli* | Tanzania | Noto | -9.89532 | 39.37409 | 277 | CDB | 2013 | NHM |
| T3218 | CB 13.241 | *Hyperolius mitchelli* | Tanzania | Noto | -9.89532 | 39.37409 | 277 | CDB | 2013 | NHM |
| T3282 | CB 13.289 | *Hyperolius mitchelli* | Tanzania | Namatimbili | -9.11064 | 39.23778 | 80 | CDB | 2013 | NHM |
| T3297 | CB 13.304 | *Hyperolius mitchelli* | Tanzania | Makangaga | -9.49454 | 39.29196 | 89 | CDB | 2013 | NHM |
| T3298 | CB 13.305 | *Hyperolius mitchelli* | Tanzania | Makangaga | -9.49454 | 39.29196 | 89 | CDB | 2013 | NHM |
| T3299 | CB 13.306 | *Hyperolius mitchelli* | Tanzania | Makangaga | -9.49454 | 39.29196 | 89 | CDB | 2013 | NHM |
| T3313 | CB 13.320 | *Hyperolius mitchelli* | Tanzania | Makangaga | -9.49454 | 39.29196 | 89 | CDB | 2013 | NHM |
| T3484 | CB 13.395 | *Hyperolius mitchelli* | Tanzania | Kiwengoma | -8.30435 | 38.90269 | 333 | CDB | 2013 | NHM |
| T3485 | CB 13.396 | *Hyperolius mitchelli* | Tanzania | Kiwengoma | -8.30435 | 38.90269 | 333 | CDB | 2013 | NHM |
| T3771 | CB 13.563 | *Hyperolius mitchelli* | Tanzania | Kibasila | -8.34888 | 36.2277 | 260 | CDB | 2014 | NHM |
| T3778 | CB 13.570 | *Hyperolius mitchelli* | Tanzania | Kibasila | -8.34888 | 36.2277 | 260 | CDB | 2014 | NHM |
| T3779 | CB 13.571 | *Hyperolius mitchelli* | Tanzania | Kibasila | -8.34888 | 36.2277 | 260 | CDB | 2014 | NHM |
| T3780 | CB 13.572 | *Hyperolius mitchelli* | Tanzania | Kibasila | -8.34888 | 36.2277 | 260 | CDB | 2014 | NHM |
| T3781 | CB 13.573 | *Hyperolius mitchelli* | Tanzania | Kibasila | -8.34888 | 36.2277 | 260 | CDB | 2014 | NHM |
| T4156 | CB 13.806 | *Hyperolius mitchelli* | Tanzania | Mabayani | -5.03361 | 38.92436 | 76 | CDB | 2014 | NHM |
| T4158 | CB 13.808 | *Hyperolius mitchelli* | Tanzania | Mabayani | -5.03361 | 38.92436 | 76 | CDB | 2014 | NHM |
| T4159 | CB 13.809 | *Hyperolius mitchelli* | Tanzania | Mabayani | -5.03361 | 38.92436 | 76 | CDB | 2014 | NHM |
| T4160 | CB 13.810 | *Hyperolius mitchelli* | Tanzania | Mabayani | -5.03361 | 38.92436 | 76 | CDB | 2014 | NHM |
| T4181 | CB 13.831 | *Hyperolius mitchelli* | Tanzania | Mabayani | -5.03361 | 38.92436 | 76 | CDB | 2014 | NHM |
| T4182 | CB 13.832 | *Hyperolius mitchelli* | Tanzania | Mabayani | -5.03361 | 38.92436 | 76 | CDB | 2014 | NHM |
| T5207 | SL 1145 | *Hyperolius rubrovermiculatus* | Kenya | Shimba Lodge | -4.2375 | 39.39563889 | 13.4 | BAB | 2014 | NHM |
| T5208 | SL 1186 | *Hyperolius rubrovermiculatus* | Kenya | Shimba Lodge | -4.2375 | 39.39563889 | 13.4 | BAB | 2014 | NHM |
| T5210 | SL 1204 | *Hyperolius rubrovermiculatus* | Kenya | Mukurmudzi | -4.375216667 | 39.42546667 | 61 | PKM | 2014 | NHM |
| T5212 | SL 1293 | *Hyperolius rubrovermiculatus* | Kenya | Shimba Lodge | -4.2375 | 39.39563889 | 13.4 | BAB | 2014 | NHM |
| T5214 | SL 1319 | *Hyperolius rubrovermiculatus* | Kenya | Scheldrick's Falls | -4.27553 | 39.43096 |  | BAB | 2014 | NHM |
| T6393 | SL 1422 | *Hyperolius rubrovermiculatus* | Kenya | Kivumoni | -4.216667 | 39.483333 |  | BAB | 2014 | NHM |
| T7541 | JVL732 | *Hyperolius mitchelli* | Tanzania | Nguru | -6.03589 | 37.52615 | 950 | JVL | 2023 | NHM |
| T7542 | JVL734 | *Hyperolius mitchelli* | Tanzania | Nguru | -6.03589 | 37.52615 | 950 | JVL | 2023 | NHM |
| T7543 | JVL801 | *Hyperolius mitchelli* | Tanzania | Kimboza | -7.01676 | 37.80815 | 340 | JVL | 2023 | NHM |
| T7544 | JVL802 | *Hyperolius mitchelli* | Tanzania | Kimboza | -7.01676 | 37.80815 | 340 | JVL | 2023 | NHM |
| T7547 | JVL24 | *Hyperolius mitchelli* | Tanzania | Kihansi | -8.58714 | 35.85014 |  | JVL | 2023 | NHM |
| T7549 | JVL28 | *Hyperolius mitchelli* | Tanzania | Kihansi | -8.58714 | 35.85014 |  | JVL | 2023 | NHM |
| T7550 | JVL29 | *Hyperolius mitchelli* | Tanzania | Kihansi | -8.58714 | 35.85014 |  | JVL | 2023 | NHM |
| T4844 | MTSN 7676 | *Hyperolius mitchelli* | Tanzania | Kimboza | -7.005459 | 37.80356 | 363 | SPL | 2012 | MUSE |
| T4846 | MTSN 7683 | *Hyperolius mitchelli* | Tanzania | Kimboza | -7.005459 | 37.80356 | 322 | SPL | 2012 | MUSE |
| T4849 | MTSN 7708 | *Hyperolius mitchelli* | Tanzania | Kimboza | -7.005459 | 37.80356 | 322 | SPL | 2012 | MUSE |
| T4850 | MTSN 7709 | *Hyperolius mitchelli* | Tanzania | Kimboza | -7.005459 | 37.80356 | 322 | SPL | 2012 | MUSE |
| T4937 | MTSN 9523 | *Hyperolius mitchelli* | Tanzania | Segoma | -4.98094 | 38.75825 | 173 | SPL | 2011 | MUSE |
| T4957 | MTSN 9549 | *Hyperolius mitchelli* | Tanzania | Segoma | -4.97643 | 38.7615 | 196 | SPL | 2011 | MUSE |
| T5143 | MUSE 11051 | *Hyperolius mitchelli* | Tanzania | Mngeta | -8.311798 | 36.091327 |  | MM | 2013 | MUSE |
| T5147 | MUSE 11060 | *Hyperolius mitchelli* | Tanzania | Mngeta | -8.311798 | 36.091327 |  | MM | 2013 | MUSE |
| T5148 | MUSE 11061 | *Hyperolius mitchelli* | Tanzania | Mngeta | -8.311798 | 36.091327 |  | MM | 2013 | MUSE |
| T5149 | MUSE 11062 | *Hyperolius mitchelli* | Tanzania | Mngeta | -8.311798 | 36.091327 |  | MM | 2013 | MUSE |
| T6046 | MTSN 5160 | *Hyperolius mitchelli* | Tanzania | Nguu | -5.480277778 | 37.47527778 | 1200 | SPL | 2008 | MUSE |
| T6137 | MTSN 8643 | *Hyperolius mitchelli* | Tanzania | Pare | -3.575338799 | 37.67276197 |  | MM | 2015 | MUSE |

**Table S2.** AMOVA (Analysis of Molecular Variance). Results of AMOVA tests for population clustering between 2 and 5 as suggested by Admixture, sNMF, fastStructure and PCA analyses. Different groupings of population clusters were tested with 999 randomisations of the data using the *poppr* R package (Kamvar et al. 2014, 2015), with k=3 being the most likely explanation of the data.

| K | Description | Test | Obs. | Std. Obs. | P-value | Φ_ST | p_value |
| --- | --- | --- | --- | --- | --- | --- | --- |
| 2 | Kenya - Tanzania + Malawi | within samples | 285.8211 | -14.522 | 0.001 | 0.333417 | 0.001 |
| 2 |  | between samples | 146.3572 | 12.37703 | 0.001 |  |  |
| 2 |  | between pops | 216.1708 | 23.79465 | 0.001 |  |  |
| 3 | Kenya - Tanzania – Malawi | within samples | 285.8211 | -14.522 | 0.001 | 0.338527 | 0.001 |
| 3 |  | between samples | 127.6037 | 10.89661 | 0.001 |  |  |
| 3 |  | between pops | 211.5815 | 23.77106 | 0.001 |  |  |
| 4 | Kenya - Northern Tanzania - Southern +Central Tanzania – Malawi | within samples | 285.8211 | -14.522 | 0.001 | 0.270494 | 0.001 |
| 4 |  | between samples | 99.17665 | 8.09183 | 0.001 |  |  |
| 4 |  | between pops | 142.7536 | 26.92995 | 0.001 |  |  |
| 5 | Kenya – Northern Tanzania – Central Tanzania – Southern Tanzania - Malawi | within samples | 285.8211 | -14.522 | 0.001 | 0.303135 | 0.001 |
| 5 |  | between samples | 70.95438 | 6.522901 | 0.001 |  |  |
| 5 |  | between pops | 155.1967 | 29.99262 | 0.001 |  |  |

**Table S3.** Genetic diversity estimates per population, including observed heterozygosity (H_o_), expected heterozygosity (H_e_), inbreeding coefficient (FIS), and nucleotide diversity (π). Metrics were calculated across *all sites* (all genotyped loci, regardless of whether they are variable or not) and *fixed sites* (loci where all individuals within a population carry the same allele).

|  | *all sites* |  |  |  |  | *fixed sites* |  |  |  |
| --- | --- | --- | --- | --- | --- | --- | --- | --- | --- |
| Population | H_o_ | H_e_ | F_IS_ | π |  | H_o_ | H_e_ | F_IS_ | π |
| Luwawa_Malawi | 0.00099 | 0.00062 | -0.00001 | 0.00099 |  | 0.05337 | 0.03323 | -0.00037 | 0.05319 |
| Makangala_Tanzania | 0.0022 | 0.00158 | 0.00056 | 0.00255 |  | 0.10219 | 0.07328 | 0.0258 | 0.11843 |
| Noto_Tanzania | 0.0023 | 0.00165 | 0.0006 | 0.00269 |  | 0.10681 | 0.07651 | 0.02807 | 0.12488 |
| Namatimbili_Tanzania | 0.00273 | 0.00137 | 0 | 0.00273 |  | 0.11198 | 0.05599 | 0 | 0.11198 |
| Makangaga_Tanzania | 0.00213 | 0.00159 | 0.00062 | 0.00251 |  | 0.10014 | 0.07457 | 0.02902 | 0.11826 |
| Kiwengoma_Tanzania | 0.00291 | 0.00166 | 0.00024 | 0.00307 |  | 0.11777 | 0.06738 | 0.00959 | 0.12416 |
| Kibasila_Tanzania | 0.0029 | 0.00224 | 0.00099 | 0.00352 |  | 0.15111 | 0.11684 | 0.05152 | 0.18332 |
| Mabayani_Tanzania | 0.00253 | 0.00207 | 0.00105 | 0.00318 |  | 0.12503 | 0.10221 | 0.05202 | 0.15685 |
| Kimboza_Tanzania | 0.00274 | 0.00216 | 0.00101 | 0.00337 |  | 0.12667 | 0.09985 | 0.04659 | 0.15543 |
| Segoma_Tanzania | 0.00274 | 0.00149 | 0.00013 | 0.00283 |  | 0.11616 | 0.06311 | 0.00551 | 0.11984 |
| Hondo_Hondo_Tanzania | 0.00289 | 0.00145 | 0 | 0.00289 |  | 0.12383 | 0.06191 | 0 | 0.12383 |
| Mgeta_Tanzania | 0.00296 | 0.00215 | 0.00085 | 0.0035 |  | 0.13118 | 0.09539 | 0.03787 | 0.15514 |
| Shimba_lodge_Kenya | 0.00185 | 0.00124 | 0.00033 | 0.00206 |  | 0.08086 | 0.054 | 0.01433 | 0.09008 |
| Mukurmudzi_Kenya | 0.00201 | 0.00101 | 0 | 0.00201 |  | 0.08815 | 0.04408 | 0 | 0.08815 |
| Sheldricks_falls_Kenya | 0.00195 | 0.00098 | 0 | 0.00195 |  | 0.08368 | 0.04184 | 0 | 0.08368 |
| Nguu_Tanzania | 0.00279 | 0.0014 | 0 | 0.00279 |  | 0.11936 | 0.05968 | 0 | 0.11936 |
| Pare_Tanzania | 0.00071 | 0.00036 | 0 | 0.00071 |  | 0.0343 | 0.01715 | 0 | 0.0343 |
| Kivumoni_Kenya | 0.00195 | 0.00098 | 0 | 0.00195 |  | 0.08495 | 0.04248 | 0 | 0.08495 |
| Nguru_Tanzania | 0.00233 | 0.00127 | 0.00013 | 0.00241 |  | 0.09598 | 0.05254 | 0.00525 | 0.09948 |
| Kihansi_Tanzania | 0.00298 | 0.00208 | 0.00072 | 0.00344 |  | 0.12865 | 0.09002 | 0.03097 | 0.14861 |

**Table S4.** Effective population size (*N_e_*) estimates using *momi2* (Kamm et al 2020)*.* For each population unit (i.e. the three clusters reported in the manuscript, the three subclusters in Tanzania, and unique sampling locality) the site frequency spectrum (SFS) was calculated, downprojected to maximise the number of segregating sites, and used to estimate the effective population size (*N_e_*).

| Population unit | SFS projection size | # Segregating sites | *N_e_* |
| --- | --- | --- | --- |
| ***Three main clusters*** | |  |  |
| Kenya | 4 | 805 | 1479.406 |
| Tanzania | 52 | 3752 | 5053.622 |
| Malawi | 4 | 109 | 620.576 |
|  |  |  |  |
| ***Three Tanzanian sub-clusters*** | | |  |
| Northern_Tanzania | 16 | 2487 | 1727.481 |
| Central_Tanzania | 12 | 1822 | 2237.325 |
| Southern Tanzania | 14 | 2196 | 826.7338 |
|  |  |  |  |
| ***Per locality*** |  |  |  |
| Shimba_Lodge | 4 | 662 | 372.261 |
| Kivumoni | 2 | 321 | 72.21515 |
| Mukurmudzi | 2 | 311 | 860.774 |
| Sheldricks_falls | 2 | 335 | 346.1901 |
| Pare | 2 | 73 | 159.3544 |
| Mabayani | 6 | 1465 | 474.0218 |
| Segoma | 2 | 505 | 147.3966 |
| Nguru | 2 | 406 | 300.0479 |
| Nguu | 2 | 348 | 349.3758 |
| Kimboza | 4 | 1012 | 492.0725 |
| Kihansi | 4 | 948 | 254.8629 |
| Mngeta | 2 | 651 | 127.3634 |
| Hondo_hondo | 2 | 357 | 244.482 |
| Kibasila | 6 | 1155 | 337.3628 |
| Kiwengoma | 4 | 557 | 110.8407 |
| Namatimbili | 2 | 484 | 101.3845 |
| Makangaga | 4 | 1049 | 206.0294 |
| Noto | 4 | 996 | 376.866 |
| Makangala | 6 | 1008 | 178.1705 |
